# Stable pollination service in a generalist high Arctic community despite the warming climate

**DOI:** 10.1002/ecm.1551

**Published:** 2022-10-02

**Authors:** Alyssa R. Cirtwill, Riikka Kaartinen, Claus Rasmussen, Deanne Redr, Helena Wirta, Jens M. Olesen, Mikko Tiusanen, Gavin Ballantyne, Helen Cunnold, Graham N. Stone, Niels Martin Schmidt, Tomas Roslin

**Affiliations:** ^1^ Spatial Foodweb Ecology Group, Research Centre for Ecological Change, Organismal and Evolutionary Biology Research Programme, Faculty of Biological and Environmental Sciences University of Helsinki Helsinki Finland; ^2^ Institute of Evolutionary Biology University of Edinburgh Edinburgh UK; ^3^ Department of Agroecology Aarhus University Tjele Denmark; ^4^ Department of Ecology Swedish Agricultural University Uppsala Sweden; ^5^ Section Genetics, Ecology & Evolutionary Biology (GEE), Department of Biology Aarhus University Aarhus Denmark; ^6^ School of Applied Sciences Edinburgh Napier University Edinburgh UK; ^7^ University of Bath Bath UK; ^8^ Department of Ecoscience Aarhus University Aarhus Denmark; ^9^ Present address: Department of Evolutionary Biology and Environmental Studies University of Zurich Zürich Switzerland

**Keywords:** diptera, *Dryas*, flower visitor, phenology, pollen deposition, pollen transport

## Abstract

Insects provide key pollination services in most terrestrial biomes, but this service depends on a multistep interaction between insect and plant. An insect needs to visit a flower, receive pollen from the anthers, move to another conspecific flower, and finally deposit the pollen on a receptive stigma. Each of these steps may be affected by climate change, and focusing on only one of them (e.g., flower visitation) may miss important signals of change in service provision. In this study, we combine data on visitation, pollen transport, and single‐visit pollen deposition to estimate functional outcomes in the high Arctic plant‐pollinator network of Zackenberg, Northeast Greenland, a model system for global warming–associated impacts in pollination services. Over two decades of rapid climate warming, we sampled the network repeatedly: in 1996, 1997, 2010, 2011, and 2016. Although the flowering plant and insect communities and their interactions varied substantially between years, as expected based on highly variable Arctic weather, there was no detectable directional change in either the structure of flower‐visitor networks or estimated pollen deposition. For flower‐visitor networks compiled over a single week, species phenologies caused major within‐year variation in network structure despite consistency across years. Weekly networks for the middle of the flowering season emerged as especially important because most pollination service can be expected to be provided by these large, highly nested networks. Our findings suggest that pollination ecosystem service in the high Arctic is remarkably resilient. This resilience may reflect the plasticity of Arctic biota as an adaptation to extreme and unpredictable weather. However, most pollination service was contributed by relatively few fly taxa (Diptera: *Spilogona sanctipauli* and *Drymeia segnis* [Muscidae] and species of *Rhamphomyia* [Empididae]). If these key pollinators are negatively affected by climate change, network structure and the pollination service that depends on it would be seriously compromised.

## INTRODUCTION

Anthropogenic environmental change is modifying species and communities across the globe (Ernakovich et al., 2014; Ovaskainen et al., 2013). However, even as there has been rapid progress in understanding the consequences in terms of shifts in species distributions, we know less about the impacts on species interactions (Brondizio et al., 2019). This is an important knowledge gap because many ecosystem functions and services are sustained by such interactions. There is thus an urgent need to identify changes in interactions and their outcomes due to the warming climate.

Ecological interaction networks provide a promising basis for understanding the functional repercussions of community change (Harvey et al., 2017; Keyes et al., 2021). They offer explicit representations of the species and their interactions and allow us to observe and model how changes in species composition (McLeod et al., 2020; Simanonok & Burkle, 2014) or phenology (Burkle et al., 2013; Memmott et al., 2007) may influence changes in species interactions. One group of functionally relevant networks are those involving plants and their animal flower visitors (Bascompte et al., 2003; Latty & Dakos, 2019; Sauve et al., 2016; Tylianakis et al., 2008). Interactions between these two groups sustain the key ecosystem function of pollination (Olesen, Stefanescu, et al., 2011; Potts et al., 2010; Rodger et al., 2021; Rosas‐Guerrero et al., 2014), although not all flower visitors are effective as pollinators (Ne'Eman et al., 2010). Using interaction networks to pinpoint changes in pollination services therefore requires the addition of data that capture the functional consequences of each interaction in the network (Ne'Eman et al., 2010).

Successful pollination has several prerequisites: Plant and pollinator must co‐occur spatially and temporally, the pollinator must visit the plant, and the pollinator must successfully carry pollen and deposit it on the viable stigma of a conspecific plant. Disruption to any of these steps, for example through changes in distribution leading to a spatial mismatch, can reduce or prevent pollination (Miller‐Rushing et al., 2010; Schmidt et al., 2016). Thus, a satisfactory representation of interactions should resolve them at a level sufficient to establish that the partners meet in relevant space and time, and to evaluate impacts of climate change, we should examine how patterns of realized co‐occurrence may be changing. In practice, we should establish changes in how interactions are realized within shorter time periods, reflecting the multiple steps from visitation to pollen deposition (Baldock et al., 2011; Ballantyne et al., 2015; King et al., 2013; Ne'Eman et al., 2010).

In a previous paper, we flagged changes in the temporal overlap between plants and their pollinators as a warning sign of impending collapse of pollination services in a well‐studied site at Zackenberg, Greenland (Schmidt et al., 2016). Based on insect abundances resolved to higher taxonomic levels (mostly families) and pollen loads counted as totals (not per plant species), we detected decreasing trends in temporal overlap between plant flowering period and in the overall pollination services provided by insects. We therefore predicted that high Arctic plant–pollinator interactions may be headed toward functional collapse.

In the present paper, we revisit this initial prediction equipped with the added resolution needed to critically examine the key ideas. We report patterns of change in a geographically isolated high Arctic plant‐pollinator network over two decades, during which time‐averaged temperature in the area rose by ~1.5°C (Schmidt et al., 2016). To evaluate the resulting impacts on plant–pollinator interactions, we revisited the local plant‐pollinator network five times (in 1996, 1997, 2010, 2011, and 2016), each time reconstructing the flower‐visitor network using identical techniques. To translate variation in flower‐visitation patterns over time to variation in the pollination service and its components, we then added functional metrics capturing selected steps of pollen transport and deposition. In 2016, we quantified temporally resolved pollen‐transport networks, as well as the amounts of conspecific pollen deposited on the stigma during a single visit (single visit deposition [SVD]) by pollinators to flowers of an abundant and frequently visited plant, *Dryas*. Drawing on this compound information, we now quantify weekly changes in species interactions over the summer and estimate the consequences of these changes for pollen transport. We then use these weekly webs to estimate the amount of pollen removed from each plant by each pollinator taxon in each week and relate this to the abundance of the pollinator. Finally, we combine these networks with SVD data to show how pollinator visits correspond to pollination services in the Arctic.

## METHODS

### Study site

The study area, the Zackenberg Valley (74°30′ N/21°00′ W) is located within Northeast Greenland National Park. The site is characterized by a relatively depauperate fauna and flora. Altogether, intensive surveys over two decades have so far revealed 160 vascular plants and 403 macroscopic, terrestrial animal species (Wirta et al., 2016). Surveys of the plant‐pollinator community have focused on patches of mesic heath containing most of the common flowering plant species (45/94 flowering plant species total) at Zackenberg.

Since the establishment of the Zackenberg research station in 1996 and the initiation of an intensive monitoring program covering both the biotic (Schmidt, Hansen, et al., 2019) and abiotic (Kandrup & Iversen, 2010; Skov et al., 2019) parts of the terrestrial ecosystem, measurements at Zackenberg have revealed both high year‐to‐year variation and directional trends in weather conditions over time (for year‐specific patterns and temporal trends 1996–2014, see Kankaanpää et al., 2018). Reflecting the large interannual variation, the range in yearly mean snowmelt in the period 1996–2014 is 38 days. Compared to a snow‐free period of only around 99 days (mean in 2006–2009) and noting the importance of snowmelt for the onset of plant flowering and arthropod activity (Høye et al., 2007, 2013), this vast variation can easily mask interannual trends. Nonetheless, temperature sums accumulating up to the mean snowmelt date (18 June) have been detectably increasing by 3.7°‐days per year during the past two decades (Kankaanpää et al., 2018).

The Zackenberg Valley forms the basis for some of the classical work on flower‐pollination networks (Bascompte et al., 2003; Dupont et al., 2009; Olesen et al., 2008; Rasmussen et al., 2013). Initial descriptions point to a structure characterized by seasonal variability in network structure due to species phenophases and a tendency for most species to interact with a few generalist “core” species (Olesen et al., 2008, 2010). Among the flowering plants in this network, one taxon is quantitatively dominant in terms of both abundance and insect attraction: *Dryas* spp. (avens) accounts for some 31% of all flowers but attracts some 97% of flower visits by insects and may therefore affect the pollination success of other plant species through competition for pollinators (Tiusanen et al., 2020). A *Dryas* flower is a flat dish supporting a brush‐like style surrounded by a dense circle of anthers (Appendix S1: Section S1: Figure S2). Nectar is secreted under the circle of anthers, meaning that most flower visitors that obtain nectar will be both coated in pollen and likely to touch one or more stigmas. Given the key role of *Dryas* in the plant‐pollinator network, we selected it as the focal taxon of our functional work.

From a taxonomic perspective, it should be noted that the two dominant species of *Dryas* crossbreed where they co‐occur. Most individuals in northeastern Greenland are hybrids between the European and North American species, *Dryas octopetala* × *Dryas integrifolia* (Elkington, 1965; Philipp & Siegismund, 2003). These hybrid individuals are fully fertile and pollinator‐dependent (Tiusanen et al., 2020, 2019, 2016), but to signal the complex status of species‐level taxonomy, we will simply refer to the taxon as “Dryas.”

### Flower visitation networks

To characterize the basic features of what insect species visit what plants, we sampled flower‐visitor networks at Zackenberg in 1996, 1997, 2010, and 2011, targeting a consistent 500 × 500‐m plot of mesic heath located in the lower plateau area between the Zackenberg research station and the coastline of Young Sound (Appendix S1: Section S1: Figure S1; Olesen et al., 2008; Rasmussen et al., 2013). The data for the flower‐visitor networks in 1996–2011 were sampled by identifying all insects visiting two individuals of each plant species during a 40‐min observation on each day with fine weather during the entire snow‐free season (for full details, see Olesen et al., 2008; Rasmussen et al., 2013). This amounted to 25 days of observation in 1996 and 1997, 54 in 2010, and 52 days in 2011, reflecting all days suitable for pollinator activity in each year. Observations without definite dates (*n* = 94) were excluded since our focus is on changes in pollination through the summer.

An additional round of sampling at the same site and using a similar protocol yielded both flower‐visitor and pollen‐transport networks for 2016. In that year, we revisited the sampling site and collected both flower‐visitor and pollen‐transport data. Flower‐visitors were observed for 51 days using the same protocol as for flower‐visitor networks in the previous years. Wherever possible, flower visitors were captured and identified using DNA barcoding (Appendix S1: Section S2). Those individuals not identified based on DNA (e.g., when sequencing failed or the specimen could not be caught) were identified morphologically to the finest possible taxonomic level. To account for differences in methodology and taxonomy over time, taxon names were harmonized across data sets (Appendix S1: Section S3).

### Pollen‐transport networks

To supplement our measures of how frequently an insect taxon visits a flower (see earlier “Flower visitation networks” subsection), with a measure of how efficiently insect taxa transport plant‐specific pollen, all insect individuals sampled in 2016 were washed for pollen (for details see Appendix S1: Section S4). The pollen grains recovered were visually identified to genus and the number of pollen grains per plant genus carried by each individual insect was recorded. The flower visitors observed in 2016 include some spiders (*Xysticus* spp.) and mites (*Parasitoides* spp.); because these taxa were not recorded in any previous networks, they were omitted in 2016 (Appendix S1: Section S2).

### Network construction

Using the foregoing data, we constructed annual and weekly networks of plants and insect flower visitors for 1996, 1997, 2010, 2011, and 2016 and separately for pollen transport in 2016. The number of weekly networks varied between years according to the length of the snow‐free season (Appendix S1: Section S2: Figure S3). Each network included all plant–insect interactions observed during the focal period (year or week).

For flower‐visitor networks, interaction weights correspond to the number of times the interaction was observed during the focal period. To make full use of the extra information from the 2016 data set, we added interactions observed in the pollen‐transport networks to the flower‐visitor networks. The pollen load carried by an insect at the time of capture is the product of an unknown number of prior visits to an unknown number of individual plants, but we may assume that the insect has made at least one visit to each plant species for which pollen was obtained. We therefore conservatively added one instance of each link observed in the pollen‐transport data to the flower‐visitor webs corresponding to the capture of the pollinator. This approach neglects the possibility of secondary pollen transport as well as the possibility that many visits contributed to an insect's pollen load. We also conservatively assumed that pollen has a relatively short residence time on an insect (Morris et al., 1995) and assigned pollen‐transport interactions to the week in which the insect was captured (in the flower‐visitor and pollen‐transport networks).

For each type of network and for annual and weekly networks, we derived several measures of network structure with proposed relationships to community stability: network size (number of plants, insects, and interactions), connectance, nestedness, and modularity (Tylianakis et al., 2010; but see Payrató‐Borràs et al., 2019). The latter three properties are among the most commonly studied aspects of network structure and have all been linked to aspects of network stability (Krause et al., 2003; Thébault & Fontaine, 2010; Tylianakis et al., 2010). Connectance describes how cohesive a network is; in a network with lower connectance, a smaller proportion of the total possible number of interactions is observed. In general, highly connected mutualistic networks have lower species persistence and resilience because the effects of a disturbance are transmitted throughout the network (Thébault & Fontaine, 2010). Nestedness (NODF) measures the tendency for specialists to interact with generalists (Bascompte et al., 2003; Bastolla et al., 2009). More nested mutualistic networks are generally considered more stable as the generalist core can buffer fluctuations in abundance (Thébault & Fontaine, 2010). This means that the removal or decline of one species is unlikely to strongly affect the rest of the network. Modularity measures the tendency for species to form groups that interact most with other members of the same group. High modularity has also been associated with increased stability, as any disturbance should be contained within the module directly affected (Krause et al., 2003). However, species in the affected module may be more likely to go extinct due to strong within‐module effects. For the flower‐visitor networks, we calculated both binary and weighted versions of connectance and nestedness. Because the meaning (“currency”) of interaction strength varies among different network types, preventing a meaningful comparison between weighted measures of pollen‐transport and flower‐visitor networks, we used only binary measures for the pollen‐transport networks. All measures were calculated using the R (R Core Team, 2016) function network level from the bipartite package (Dormann et al., 2008).

### Estimation of pollination service

To convert observations of flower–insect interactions into estimates of net pollen transfer, we estimated two components of pollination service: pollen transport (for all plants) and pollen deposition (for *Dryas* only). We estimated weekly and annual pollen transport for each species based on the data used to construct the aforementioned pollen‐transport networks (Appendix S1: Section S5). For these estimates, we used the mean pollen loads per plant species recovered from each insect individual, averaged over individuals within an insect taxon (species or genus; see Appendix S1: Section S2 and S3).

Pollen deposition on *Dryas* was estimated by counting the conspecific pollen deposited on virgin *Dryas* flowers during a single visit by a pollinator (see Appendix S1: Section S6 for details). To estimate the total pollination service to *Dryas*, we multiplied the number of visits each insect taxon made to *Dryas* per week (as derived from the weekly flower‐visitor networks) by empirical estimates of the mean number of conspecific pollen grains deposited during a single visit to a virgin flower. Little pollen was found on control (unvisited) flowers (median = 2, mean = 19, SD = 45.5, *n* = 9). Where possible, we used means of single‐visit deposition data calculated at the level of insect genera. As the identity of many of the flower visitors observed during the SVD sampling remained taxonomically poorly resolved, we used mean pollen deposition per insect family where genus‐level data were not available.

Finally, we compared the estimated total amount of *Dryas* deposited by each insect to the length of its activity (flight) period (Appendix S1: Section S9). Insect flight periods are generally stable across years (Schmidt et al., 2016). To avoid potential errors due to missing the start or end of flight in any given year, we define each insect's active period as the average number of weeks in which insects were observed in each year.

### Statistical analyses

#### Comparing network structures

To examine whether observed networks significantly differed between years, we compared observed metrics of network structure to distributions of values derived from random sampling of the 5‐year metaweb. We drew 1000 same‐size random networks for each annual network and then calculated the same set of network structure metrics for the random and observed networks. Each random network had the same number of plants and insects as the observed networks. Each plant–insect pair in the random network was assumed to interact if the pair interacted in the metaweb; interaction strengths were drawn from a uniform distribution spanning the range of observed interaction strengths. The network structures of the random networks were then used as a null distribution for each property. An observed network had significantly nonrandom structure if it was lower than the 0.025 quantile or exceeding the 0.975 quantile of the null distribution.

Applying the same logic, we compared structural metrics of weekly networks to distributions of metrics based on 1000 random draws from the corresponding annual network to test whether network structure varied significantly within a year. Random networks for each weekly network were constructed as previously described, preserving numbers of plants and insects but allowing numbers of interactions to vary depending on the species selected. Again, we drew interaction strengths from a uniform distribution spanning the observed strengths. Observed values lower than the 0.025 quantile or exceeding the 0.975 quantile of the expected distribution were deemed statistically significant.

Finally, to evaluate whether structural descriptors based on flower visits and pollen transport painted a consistent picture of variation in network structure over time, we calculated both Pearson and Spearman (rank‐based) correlations (ρ) for each network property between network types. Each analysis was conducted for network metrics: the number of plant taxa in the network, the number of insect taxa, the number of links, binary connectance, binary NODF, and the value of modularity (described earlier, see “Network Construction”).

#### Relating different steps in pollination

Turning from network structure to estimated total pollination service to *Dryas*, we tested for relationships between pollen deposition and other aspects of pollination. First, we tested whether insect genera that deposit more *Dryas* pollen in a single visit either tend to carry more pollen in general or tend to make more visits by fitting two linear models. We regressed mean SVD per genus against mean total pollen on an insect and mean total observed visits to *Dryas* in a single year. Next, we tested whether total estimated conspecific pollen deposition on *Dryas* was associated with the length of an insect taxon's activity period. We fit a linear model relating the estimated deposition per taxon per year to the taxon's activity period, year, and their interaction. We then simplified the model by removing nonsignificant interaction and main‐effect terms, leaving a model relating pollen deposition to activity period only. All regressions were fit using the R (R Core Team, 2016) base function lm. In addition, we calculated correlations corresponding to each regression using the R (R Core Team, 2016) base function cor.

## RESULTS

**FIGURE 1:**
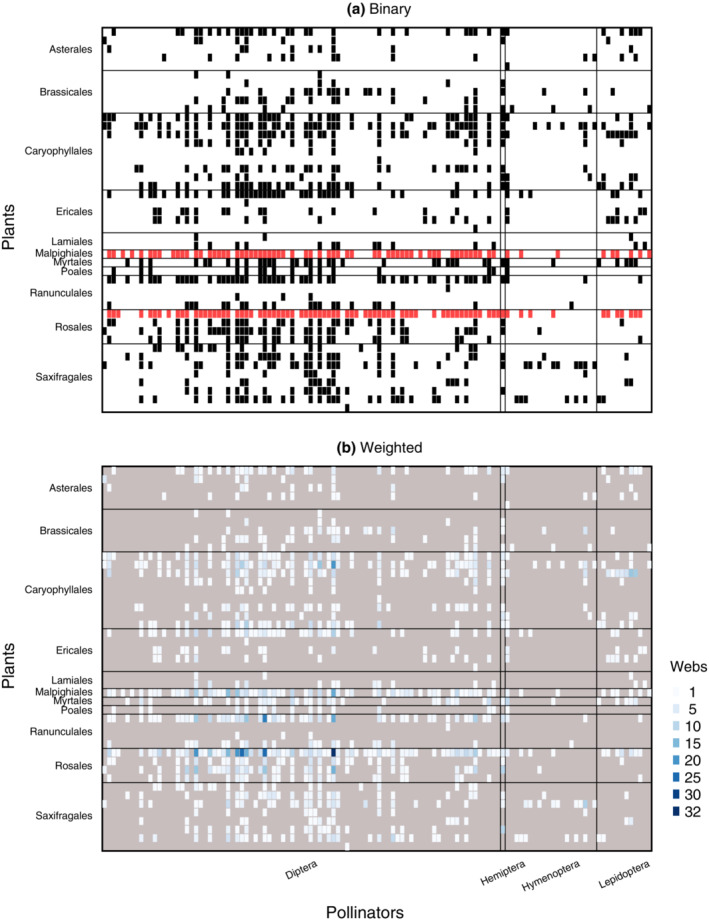
The Zackenberg plant‐pollinator network compiled across 5 years of observations shows that only a small fraction of all possible interactions have been realized. Each species of plants and pollinators is shown as a line or a row within its order. (a) In the binary metaweb combining observations from flower‐visitor networks and pollen‐transport networks across all 5 years, most pollinators visited two plants (*Dryas*; Rosaceae, Rosales and *Salix arctica*; Salicaceae, Malpighiales, highlighted in red). (b) Many interactions (498/990) were observed in only one weekly network, with only seven interactions observed in 20 or more weeks across the 5 years considered. In the weighted metaweb, cell colors indicate the number of weekly networks including the focal observation. The darker the cell, the more often the interaction was observed. Missing interactions are indicated in gray to increase the visibility of rarely observed interactions.

**FIGURE 2:**
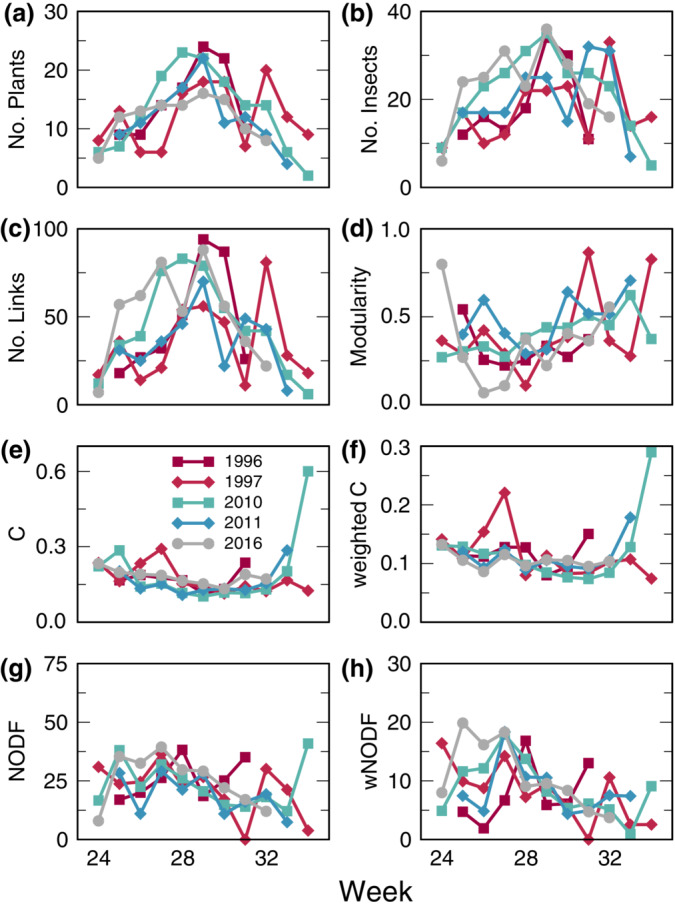
The structural properties of the weekly flower‐visitor networks showed substantial consistency across the 5 years of observations. (a–c) Numbers of plants, insects, and links observed per week all peak midseason and were similar across years. (d) Modularity was lowest in midseason but varied considerably across years. (e, f) Binary and weighted connectance (c) are both generally lower midseason and were similar across years. (g, h) Binary and weighted nestedness (NODF) generally declined over the course of the summer but were highly variable between years.

In the high Arctic plant‐pollinator network of Zackenberg, the realized interactions (links) occurring in any given time period are only a small fraction of the possible interactions (Figure 1 and Appendix S1: Section S2: Figures S3 and S4). More specifically, with a connectance of 0.183, only about one in five possible links was realized in the 5‐year metaweb. The average connectance in the weekly networks was similar to the connectance of the annual web, although connectance varied widely between weeks (range 0.103–0.600, mean = 0.213, median = 0.182; Figure 2). Here, the relatively high connectance within some weekly networks can partly be attributed to the fact that in calculating this value, we only include the species occurring during the respective week, not all species present in the system over the entire season. Unsurprisingly, the highest connectance occurred in a network with only two plants; the requirement that all pollinators visit at least one of these plants trivially implies a minimum connectance of 0.5. Interactions cannot occur between species that do not temporally co‐occur, and these absent species and “forbidden links” should be excluded when calculating network structure (Olesen, Bascompte, et al., 2011).

### Flower‐visitation and pollen‐transport networks have similar structures

**FIGURE 3:**
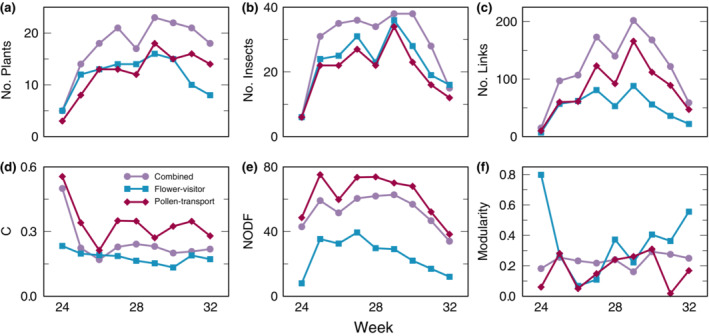
The structural properties of weekly networks in 2016 differed depending on the sampling methodology: flower visitation only, pollen transportation only, or both. Network properties are as in Figure 2. (a–c) The flower‐visitor and pollen‐transport networks contained similar numbers of species, but there was not complete overlap between data sets. The pollen‐transport network contained substantially more links than the flower‐visitor networks for most weeks. (d–f) The pollen‐transport networks were more connected and more nested than the combined network, but less modular. In the combined network, modularity was nearly constant throughout the summer.

Compared to insect–plant networks reconstructed from visitation patterns, pollen‐transport networks—that is, interaction networks inferred from the pollen loads carried by individual pollinators—tended to have higher connectance and higher nestedness (Figure 3; for test statistics see Appendix S1: Section S7). Because pollen was not recovered from all flower visitors, the network combining the flower‐visitor and pollen‐transport networks for 2016 contained more plants, insects, and links than either network type on its own. Pollen‐transport and flower‐visitor data therefore complement each other to more fully describe the system. However, this summary network had similar structural properties (e.g., nestedness, modularity) to each of its two component networks alone (Tables S2–S4, for test statistics see Appendix S1: Section S7). Thus, although flower‐visitor and pollen‐transport networks each contain some unique species and interactions, they provide qualitatively similar insights into the structure of the community.

### Networks are strongly structured by phenology

Most plants, insects, and interactions were observed in the middle of the flowering season, while connectance was lowest midseason (Figure 2 and Appendix S1: Section S2: Figure S3). This indicates that many species with midseason activity are relatively specialized with few interactions. Structural metrics for weekly networks were significantly different from expectations based on random realizations of the annual networks according to many measures of network structure (*p* < 0.05; Appendix S1: Section S8: Table S5). This pattern results from strong phenological structure in the pools of active species that are available to interact. However, such variation in who interacted with whom did not drive similar variation in the seasonal progression of network structure between years (Figure 2, Appendix S1: Section S7).

### Functional consequences of phenological patterns

**FIGURE 4:**
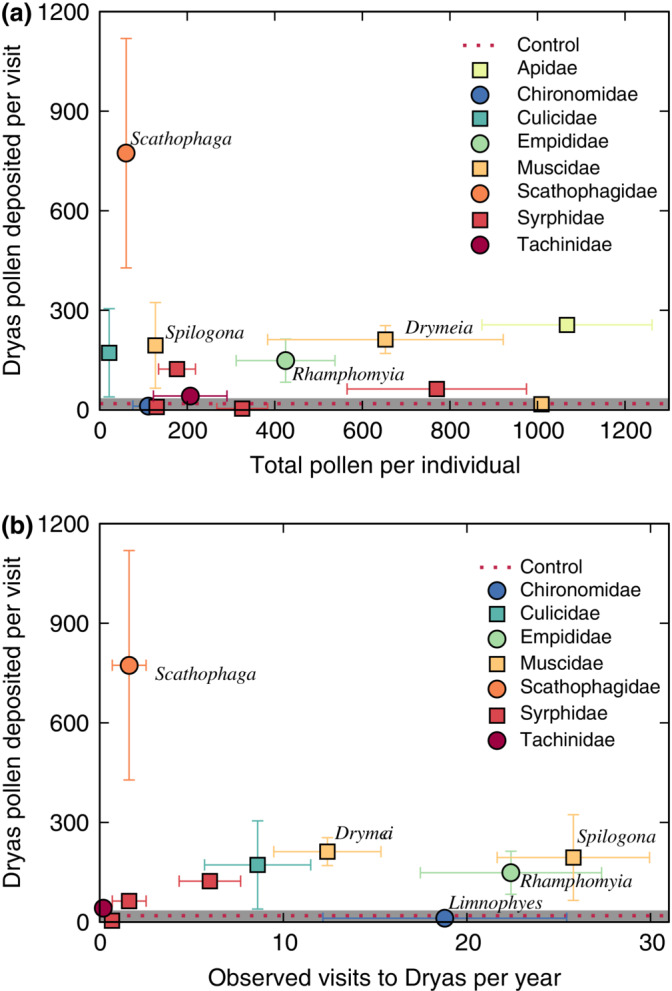
Number of conspecific pollen grains deposited on a *Dryas* flower during a single visit deposition (SVD) was similar across most genera. (a) Mean SVD was not related to mean total pollen on an insect's body (*R* ^2^ = 0.080, β = −0.307, *p* = 0.778). (b) Mean SVD was not related to mean annual visits to *Dryas* (*R* ^2^ = −0.20, β = −30.2, *p* = 0.472). SVD values are shown for genera for which SVD data are available and that were observed visiting *Dryas*. Error bars indicate ±SE. Mean pollen observed on nine control (unvisited) *Dryas* flowers is indicated by the dotted red line (standard error [SE] shown by gray shaded bar).

**FIGURE 5:**
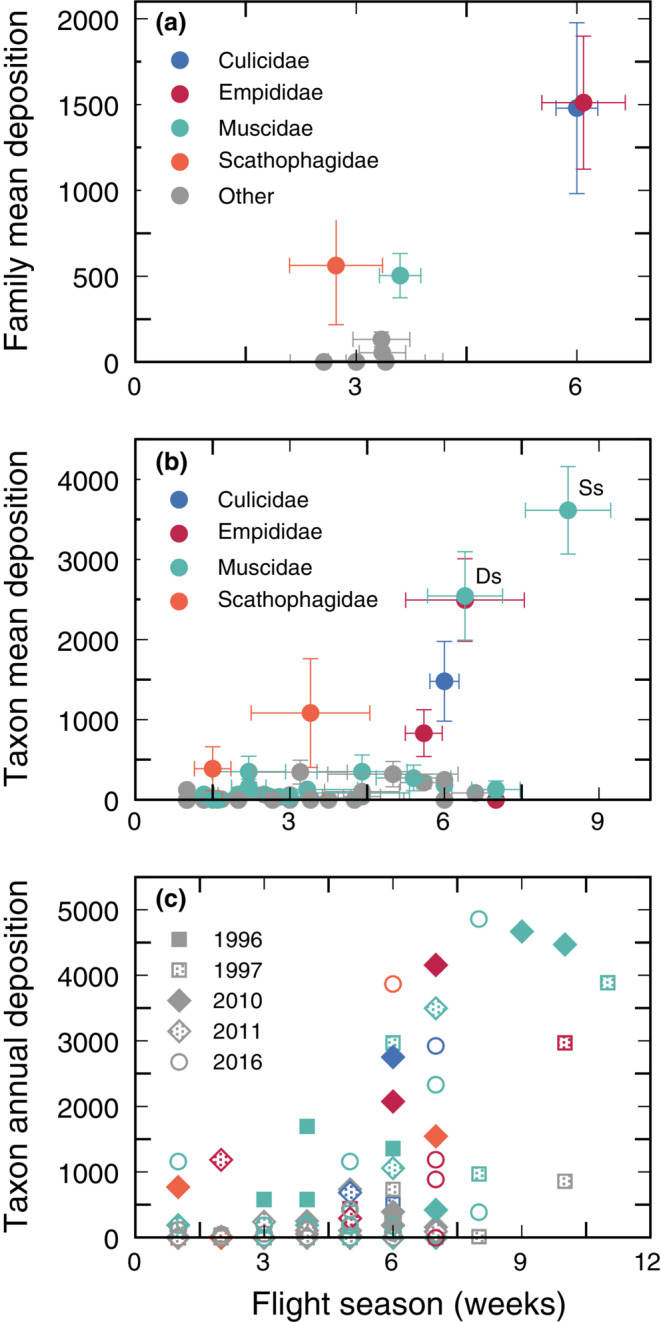
Annual estimated pollination service to *Dryas* tended to increase with the length of an insect's activity period, whether considered at the family level across years, taxon level across years, or within a single year. (a) Mean activity periods for insects in the families Culicidae and Empididae were several weeks longer than the mean activity periods for other families. The mean total annual pollen deposition for insects in these families was also substantially higher than in other families. Each point represents the mean of all annual totals for all insect taxa within a family (usually species) across all years in which the insect was observed. Error bars indicate ±1 SE. (b) Mean total annual pollen deposition for a single insect taxon across years also tended to increase with increasing mean activity period across years, although there was substantial variation within the Muscidae. Two taxa (*Drymeia segnis* and *Spilogona sanctipauli*, indicated by Ds and Ss, respectively) were expected to transport much more pollen than other muscids. The Culicidae were represented by a single taxon (*Aedes* sp.). Each point represents the mean of all annual totals for an insect taxon (usually species) across all years in which the insect was observed. Colors indicate family as in (a); error bars indicate ±1 SE. (c) There was no obvious difference across years in the relationship between pollen deposition and insect activity periods over time. Each point indicates the estimated total *Dryas* pollen deposition for an insect taxon (usually species) in a single year. Colors indicate family as in (a). Symbol shape and fill indicate year. SE, standard error.

Body pollen load size varied substantially among insect taxa (Appendix S1: Section S5: Figures S5, S7), but the size of the conspecific pollen load deposited on a stigma during a visit did not reflect this variation (*R* ^2^ = 0.080, β = −0.307, *p* = 0.778; Figure 4). For example, *Bombus* had body pollen loads five times greater than *Spilogona*, but each deposited similar numbers of pollen grains on a single visit. However, pollen deposition increased significantly with the length of an insect's active period (*R* ^2^ = 0.567, β = 224, *p* < 0.001; Figure 5). Thus, except for very poor pollinators such as *Limnophyes*, an insect taxon's contribution to *Dryas* pollen deposition was more dependent on the total number of visits by the taxon (based on abundance, length of active season, and so forth) than on finer variation in, for example, pollen‐deposition behavior.

**FIGURE 6:**
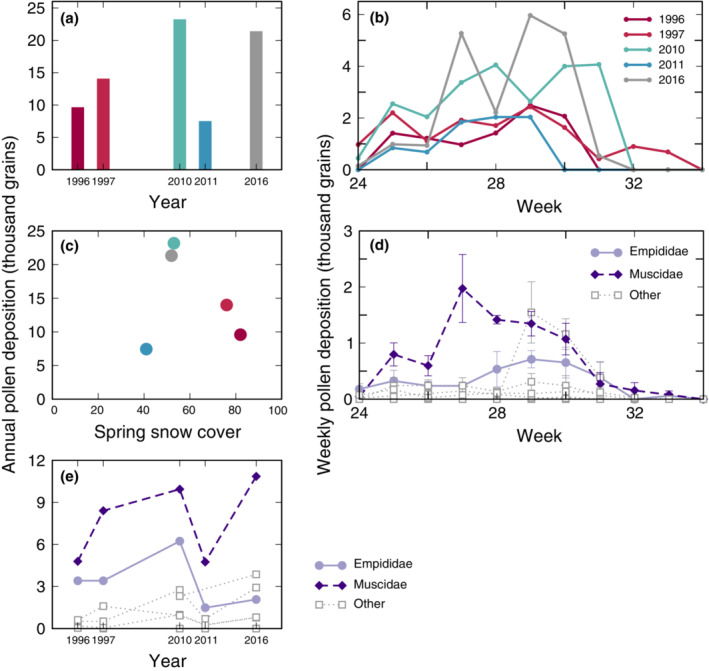
(a) Estimated total pollination service to *Dryas* was variable across years but did not show a clear trend over the 20 years considered in our data set. Pollination service was estimated by multiplying the total number of visits to *Dryas* by the mean number of conspecific pollen grains deposited on the stigma in a single visit, summed over all insects for which single visit deposition (SVD) pollen deposition was observed. (b) Pollen deposition strongly peaked midseason in 2010 and 2016 but was more consistent across weeks in other years. We show the estimated total number of pollen grains deposited by all insects in each week in each year. Line color indicates year. (c) Total pollen deposition on Dryas plotted against spring snow cover (second week of June; taken from Schmidt, 2019). (d) Mean total pollen deposition per insect family per week. Empididae (solid line) and Muscidae (dashed line) deposited more pollen in most weeks than other families (dotted lines). Empididae and Muscidae are therefore highlighted in purple in panels (d) and (e). Each line represents the mean of total pollen deposition for an insect family across the 5 years in our data set. (e) Total pollen deposition in all years was dominated by insects in the families Empididae and Muscidae. We show total pollen deposition by insects within each well‐documented family in each year. Totals are sums across all taxa within a family (usually species) for which pollen deposition could be estimated. Line color and style indicate family as in (d).

The summed effects of year‐to‐year variation in which insects and plants meet, what pollen is transported between plants, and what pollen is deposited on stigmas results in substantial variation in the year‐specific mechanics of plant pollination at Zackenberg (Figure 6; we refrain from formal tests since we have only 5 years of data). Years with especially high total pollen deposition also showed strong peaks in weekly deposition in midsummer, highlighting the importance of this period. Lower spring snow cover appears to be associated with greater pollen deposition, except for the year with least snow (2011), which was particularly dry (T. Roslin and N. M. Schmidt, personal observation). Greater total pollen deposition is also associated with greater deposition by empidid and muscid flies, reinforcing the key role of these taxa as Arctic pollinators.

## DISCUSSION

Prior warnings that climate change may collapse the interaction networks that provide pollination service in the high Arctic (Høye et al., 2021; Høye & Forchhammer, 2008; Loboda et al., 2018; Schmidt et al., 2016; Tiusanen et al., 2020) have, by necessity, been based on some leaps of faith from pattern to process. After filling in key gaps along the inference chain, we now observe remarkable resilience in the structure of highly resolved plant‐pollinator networks spanning two decades, and in the likely total pollen transfer resulting from all interactions within annual webs. In terms of the structure of the annual webs, we had expected climate change to cause consistent, directional changes from 1996 to 2016. Instead, annual networks were consistent with random sampling from the 5‐year set of interactions and had consistent structure between years. While we did observe expected variation in weekly network structure over the course of the summer, these intra‐annual patterns were also consistent between years.

As well as comparing network structure over time, we were also able to estimate the total pollen transport service to a key plant (*Dryas*). Here, we found that the resilience observed in terms of network structure corresponds to functional resilience in terms of pollen transport. Summed estimates of (*Dryas*) pollen moved to and deposited on conspecific flowers showed no directional change between years. These results have important implications for how we interpret the effects of climate change on ecological networks and on the ecosystem services they provide.

### Arctic plant‐pollinator networks seem resilient over time

Whether considered at an annual or weekly level, we did not observe any directional change in the structure of plant‐pollinator networks at Zackenberg over two decades. This contrasts with our earlier predictions for the system (Cirtwill et al., 2018; Schmidt et al., 2016), which suggested that declines in insect populations could lead to the collapse of plant–pollinator interactions and an unraveling of the network (i.e., loss of connectance and other structural features or breaking into smaller components). We did find substantial differences in the set of species observed each year (although not significant: *F*_1,3_ = 0.798, *p* = 0.733 for a permutational multivariate analysis of variance (PERMANOVA) based on 119 permutations and one sample per year). Unsurprisingly, there were also differences in the interactions observed each year (Appendix S1: Section S2: Figure S3); however the consistency of structural measures suggests that these changes reflect interaction rewiring rather than a gain or loss of species over time. In a network context, rewiring can arise through plasticity in the abilities of specific plant and insect taxa to interact, allowing retention of species in a network through flexibility in the partners they depend upon. Such rewiring is expected to play a key, though underestimated, role in maintaining community structure and stability (CaraDonna et al., 2017; Olesen, Stefanescu, et al., 2011; Staniczenko et al., 2010). In systems like Zackenberg, where many species have generalist morphology (Olesen et al., 2008), there should be few obstacles to widespread rewiring compared to systems in which links are constrained by morphology or other traits (for an opposite example, see, e.g., Davis et al., 2015).

In addition to a high potential for rewiring, structural resiliency in the Zackenberg system is likely driven by a few key taxa. Among the plants, *Dryas* and *Salix arctica* stand out for their large number of interaction partners (Figure 1). These plants have long flowering periods (Appendix S1: Section S9: Figure S8) and are individually long‐lived. *Dryas* may live for over a century (Elkington, 1971), and *S. arctica* may live for over 200 years (Flora of North America Editorial Commitee, 2010; Schmidt et al., 2006). Because the high Arctic is characterized by famously variable weather (Blix, 2007; Schmidt, Reneerkens, et al., 2019), such long‐lived plants may have evolved to tolerate a range of conditions, which largely includes current levels of climate change (Landrum & Holland, 2020). For these plants, evolving traits to maximize a single season's reproductive output may be less important than maintaining traits that provide access to a large pool of different pollinators, allowing the opportunity for pollination and reproduction under a wide variety of conditions. The latter strategy may then secure reliable pollination success, maximizing seedling access to ephemeral gaps in otherwise dense Arctic vegetation (T. Roslin and N. M. Schmidt, personal observation).

Among insects, the key pollinators were muscid flies (Muscidae) and dagger flies (Empididae). Both groups have relatively long flight periods (Appendix S1: Section S9: Figure S8) and carry large amounts of pollen (Figure 4 and Appendix S1: Section S5: Figures S5, S7). Pollinators with long active periods at Zackenberg tend to be highly abundant and visit many plant taxa, becoming core components of the network (Rasmussen et al., 2013). Part of this generality is likely to be necessary, since species with long activity periods must find nectar resources throughout their active season (Baldock et al., 2019). The same plasticity may also buffer these species against changes in the abundance of any particular plant. However, the key role of particular insects also highlights a potential vulnerability in the system: If the Muscidae and Empididae were to decline and no new pollinators visited *Dryas* in sufficient numbers to replace them, then pollination would be greatly reduced. In our networks, all Empididae belonged to a single genus (*Rhamphomyia*; five species present in Greenland; Böcher et al., 2015), whereas the most effective pollen transporters among the Muscidae were *Drymeia segnis* and *Spilogona sanctipauli*. Thus, despite the diversity of Muscidae at Zackenberg (Tiusanen et al., 2016; Wirta et al., 2016), in ecosystem function terms the impact of both Muscidae and Empididae rests upon a small group of species.

Based on their key role as pollinators, we might expect that any decline in Muscidae would be associated with a decline in pollination service. However, although it has recently been suggested that muscid densities have declined by ~80% at Zackenberg from 1996 to 2014 (Loboda et al., 2018), we did not observe a corresponding decline in muscid visits to our focal plant, *Dryas*. How are we to reconcile these contradictory findings?

First and most importantly, the decline previously reported is habitat and site‐specific. The plots used for monitoring arthropod densities at Zackenberg were originally selected to represent different habitats (Appendix S1: Section S1: Figure S1; Schmidt, Hansen, et al., 2019), meaning that abundance trends may not apply to the mesic heaths we consider here. Indeed, recent work reveals varied population trends in different environments (Loboda et al., 2018), with inconsistent support for population declines from a recent reanalysis (Høye et al., 2021).

If insect abundance is truly declining, these declines appear largest in wet fens (Høye et al., 2021; Loboda et al., 2018). Because these habitats are dominated by grasses and mosses rather than insect‐pollinated plants, they were not included in our pollination network sampling (Olesen et al., 2008). In mesic heaths, on the other hand, muscid abundances were more stable over time (Loboda et al., 2018). Moreover, although muscids overall declined in wet fens, the key muscids for *Dryas* pollen transport (*S. sanctipauli* and *D. segnis*) did not show changes in abundance in mesic heaths (Høye et al., 2021; Loboda et al., 2018). Although such a large decline in muscid abundance near our study site is worrying, the more constant abundances in our focal habitat mean that any effect on the mesic heath pollination networks should so far be small.

Second, as emphasized earlier, these key flies are active for a long period (Appendix S1: Section S9: Figure S8) and carry large numbers of pollen grains (Appendix S1: Section S5: Figure S7). These are the very characteristics that make them highly effective pollen transporters on a weekly and yearly basis. Phenological shifts and declining abundances during some part of the flight season may thus be counterbalanced by constant or increasing abundances during others, especially for plants such as *Dryas* that have long flowering periods.

Third, since *Dryas* is an abundant and highly competitive plant (Tiusanen et al., 2020), it is possible that small declines in Muscidae might not affect our estimates of pollination service. The plants most likely to be affected by a small decline are the rarest and least‐attractive plants, for which each visit by a potential pollinator is likely to matter intensely (Dakos & Bascompte, 2014). The ecological networks we present are comparatively large and well sampled (83–108 species and 244–389 interactions) but nevertheless only capture a small subset of the interactions that actually occur. Like all network data, they are particularly biased against including interactions among rare taxa (Cirtwill et al., 2019), making it easy to miss signals of change in how these rare species fit into their community. Although we focused on *Dryas* because of its key role in the Zackenberg pollination system, future work aiming to identify early signs of declining ecosystem service should focus on rarer taxa. With targeted sampling, it is possible to overcome the bias against rare species and interactions and more accurately detect any decline in pollination service (Cirtwill et al., 2019; Dakos & Bascompte, 2014).

## CONSTRUCTING MORE INFORMATIVE NETWORKS

### Greater temporal resolution reveals important within‐year trends

Our interaction networks are highly resolved within a year, allowing us to construct weekly and annual networks. This allows us to suggest more and less sensitive periods within the pollination season. Overall, the Zackenberg plant‐pollinator community is sparsely connected, but this aspect displays considerable variation within a summer. Most interactions occur in the middle of the flowering season, leading midseason networks to be less modular and slightly more nested than early‐ or late‐season networks (Figure 3). These structural properties suggest high stability and resilience during this midseason period (Tylianakis et al., 2010).

Moreover, the observed and expected phenological shifts in plant and pollinator activity periods (Høye et al., 2013; Schmidt et al., 2016) are most likely to affect interactions occurring at the beginning and end of species' active periods. The midseason community is less likely to have been affected by major phenological shifts so far. The early‐ and late‐summer networks that might show signals of such phenological shifts are also the most variable between years (Figure 2), making it difficult to tease apart effects of climate change from the intrinsic variability of the system (Landrum & Holland, 2020). If phenological shifts continue their past trajectory, however, we will observe disruption to this midseason core community in the coming years. Similarly, we can predict that midseason disturbances, such as storms, frosts, and heat waves, will have sizeable effects since they will affect the large midseason active community. Because increasingly frequent severe weather is also a predicted consequence of climate change (Masson‐Delmotte et al., 2021; Ornes, 2018), it is important to collect short‐term interaction data to match these short‐term disturbances.

### Combining network types suggests constant stability

Having collected visitation and pollen‐transport data in 2016, we are also able to clarify how methodological choices may affect network structure. The pollen‐transport network included many more species and interactions than the visitation network, consistent with other comparisons of sampling methods (Bosch et al., 2009). However, both sets of networks showed similar structural patterns within the year (Figure 3). This may be because Zackenberg is a relatively species‐poor and generalist community, such that different sampling methods are likely to capture similar pools of species. These similar annual patterns suggest that visitation and pollen‐transport networks may safely be combined in future analyses that focus on within‐year changes—after accounting for size‐dependent changes in network structure (Martinez, 1993; Martinez et al., 1999).

Because the plant‐focused visitation networks and pollinator‐focused pollen‐transport networks did record different species and interactions (indicated by the larger size of the combined networks in Figure 3), the best approach would be to sample using both methods. Combining multiple sampling methods in a consistent way reduces bias toward particular taxa (Gibson et al., 2011; Jordano, 2016) and increases the total sample size, meaning that the combined networks are more likely to reflect the true community (Blüthgen et al., 2006; Cirtwill et al., 2019). In our system, we see that the combined network has more consistent connectance, nestedness (particularly binary nestedness), and modularity throughout the year than either network type considered separately. Because these structural properties are commonly linked to community stability (Tylianakis et al., 2010; but see Payrató‐Borràs et al., 2019), the Zackenberg community could be similarly stable throughout the year. This consistency would not be apparent if we had access to only one type of data.

### From network structure to service delivery

Ultimately, testing an earlier prediction of the imminent collapse of pollination service in the Arctic (Schmidt et al., 2016) requires us to unite networks with data that capture other steps in the pollination process. To estimate the amount of pollination service provided by a focal pollinator to a focal plant, both the number of visits and number of conspecific pollen grains deposited on the stigma per visit are required (Alarcón, 2010; Bosch et al., 2009; Popic et al., 2013). Combining these different sources of information, we can examine not only total pollination service but also how this service accumulates through time and how different pollinator taxa contribute to the net pollination of particular plant species.

## CONCLUSIONS

In summary, our study increases the calls for adding functional perspectives to understand changes in plant–pollinator interactions. Pollination is a key ecosystem function and has been intensively studied over recent decades (Olesen et al., 2010; Potts et al., 2010; Rosas‐Guerrero et al., 2014). Relating the structure and functionality of pollination networks is, however, something that has rarely been attempted (Ballantyne et al., 2015; Ferrero et al., 2013; Kaiser‐Bunbury et al., 2010). Current knowledge on plant–pollinator interactions is largely based on patterns in flower visitation, despite the awareness that this gives an overly generalized view of pollination. Thus, comparative studies relating observed visitation networks to functional differences between species are needed to revise our understanding of the properties of plant–pollinator interactions. The current study reveals how this information can be used to model and predict how networks react to perturbations caused by global change. It suggests that, although the plant‐pollinator networks of the high Arctic are already subject to major environmental change (Landrum & Holland, 2020), functional impacts may so far have been buffered by the resilient features of the system. Because insect‐pollinated plants are globally important for maintaining biodiversity and human food sources (Klein et al., 2007; Ollerton et al., 2011; Rader et al., 2016), similar analyses in other systems have an obvious appeal. For the Arctic system, the current analyses are some of the only ways to explore functional consequences before the *fait accomplit*. Given the long generation time of *Dryas* (Elkington, 1971), actual population‐level effects of changes in plant‐pollinator networks are likely to be slow. Thus, once shrinking populations of key plants are obvious, the actual pollination service that originally allowed them to establish could be long gone.

## AUTHOR CONTRIBUTIONS

Jens M. Olesen, Claus Rasmussen and Riikka Kaartinen led the characterization of the system in 1996–1997, 2010–2011, and 2016, respectively, upon which the work was based. Alyssa R. Cirtwill and Tomas Roslin led the synthesis of interaction dynamics at different levels over time. Alyssa R. Cirtwill led the statistical analysis of network structure. Helena Wirta led the identification of samples through molecular techniques, and Mikko Tiusanen conducted the microscopy‐based quantification of pollen loads. Gavin Ballantyne and Helen Cunnold generated the empirical data on SVD, and Deanne Redr led their analysis. Graham N. Stone assisted in collection of field data in 2016, and Niels Martin Schmidt conducted the biomonitoring at Zackenberg of which this initiative is a part. All authors contributed to the writing and revision of the manuscript.

## FUNDING INFORMATION

Tomas Roslin was funded by the European Research Council (ERC) under the European Union's Horizon 2020 research and innovation program (Grant Agreement 856506; ERC‐synergy project LIFEPLAN), by the Academy of Finland (Grant 322266), and by the Jane and Aatos Erkko Foundation.

## CONFLICT OF INTEREST

The authors declare no conflict of interest.

## Supporting information


Appendix S1

## Data Availability

The sequence data sets generated in the current study are available in GenBank under accession numbers MZ428281–MZ429016 and in the Sequence Read Archive under BioProject PRJNA748227 at https://www.ncbi.nlm.nih.gov/bioproject/PRJNA748227.
