# Supplementary material for: Stable pollination service in a generalist high Arctic community despite the warming climate
Source: Ecol Monogr. 2022 Oct 2;93(1):e1551. doi: 10.1002/ecm.1551 (PMC10078371; doi:10.1002/ecm.1551)
Supplement: Supplementary file 1 — Appendix S1 [file ECM-93-0-s001.pdf]

Stable pollination service in a generalist High Arctic community despite the warming climate

Ecological Monographs

Alyssa R. Cirtwill[1†*], Riikka Kaartinen[1,2*], Claus Rasmussen[3*], Deanne Redr[4*], Helena Wirta[1*], Jens M. Olesen[5], Mikko Tiusanen[1,6], Gavin Ballantyne[7], Helen Cunnold[8], Graham N. Stone[2], Niels Martin Schmidt[9], Tomas Roslin[1,4]

[*] In alphabetical order, equal contribution

[1] Spatial Foodweb Ecology Group, Research Centre for Ecological Change, Organismal and Evolutionary, Biology Research Programme, Faculty of Biological and Environmental Sciences, University of Helsinki

[2]Institute of Evolutionary Biology, University of Edinburgh, Edinburgh, EH9 3FL, UK, [3]Department of Agroecology, Aarhus University, 8830 Tjele, Denmark, [4] Department of Ecology, Swedish Agricultural University, 75007 Uppsala, Sweden, [5]Section Genetics, Ecology & Evolutionary Biology (GEE), Department of Biology, Aarhus University, 8000 Aarhus C, Denmark, [6]Current address: Department of Evolutionary Biology and Environmental Studies, University of Zurich, Zürich, Switzerland, [7]School of Applied Sciences, Edinburgh Napier University, [8]University of Bath, BA2 7AY Bath, UK, [9] Department of Ecoscience, Aarhus University, Aarhus, Denmark

[†] Corresponding author: alyssa.cirtwill@gmail.com

Section S1 Description of study site

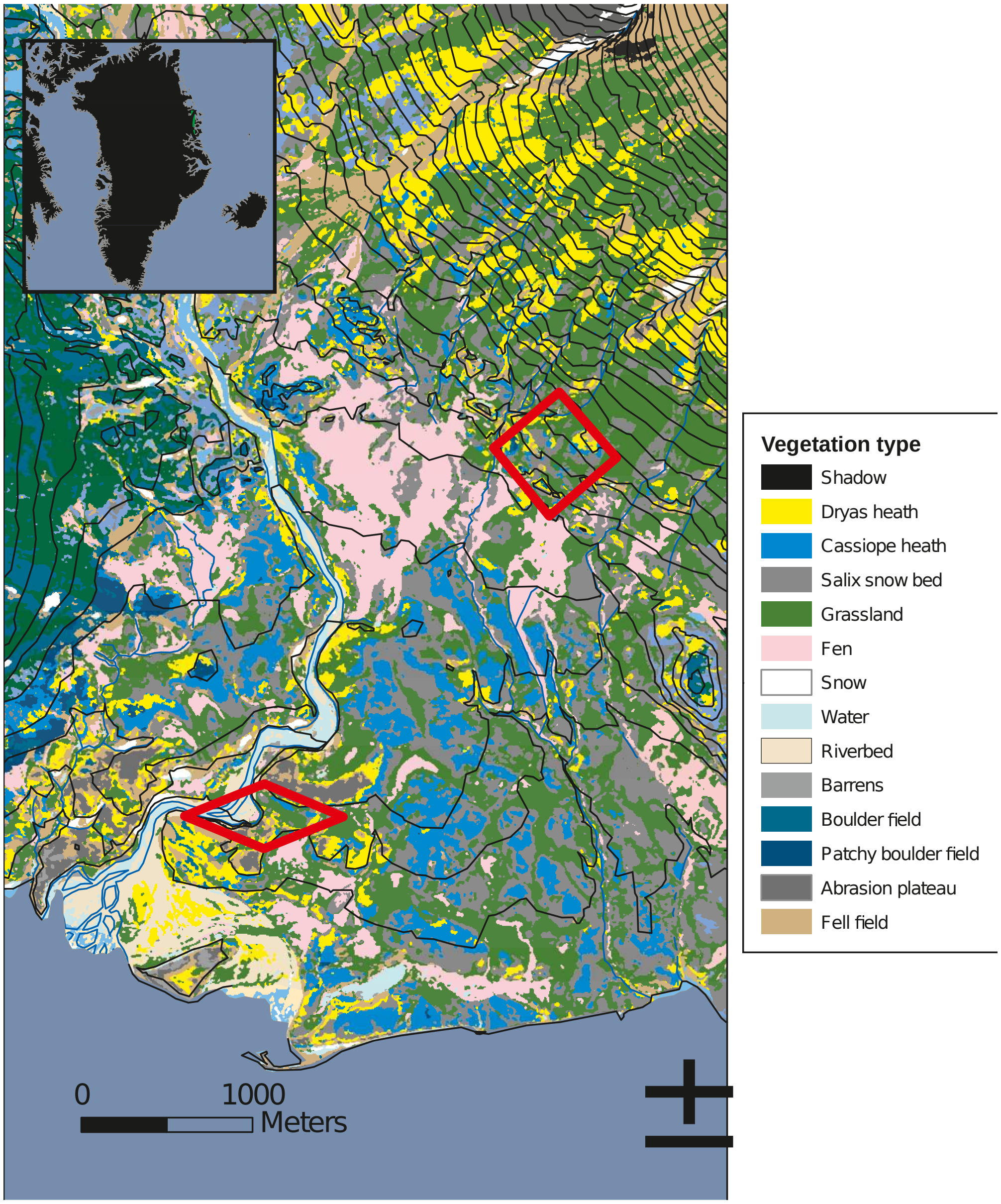

Figure S1: Map of Zackenberg valley with elevation gradients and vegetation types (indicated by colours), showing the study plots (red diamonds). Plot 1 is located to the south and plot 2 to the north. Figure kindly provided by Jannik Hansen.

The plant-pollinator community at Zackenberg (Fig. S1) is highly isolated from potential insect colonists. First, the distance between Greenland and either mainland Europe or North America is long and difficult for insects or seeds to traverse. Further, it is important to distinguish between Western and Eastern Greenland. These two regions are, for the most part, separated by a glacier with a width of 500-1000 km, offering a formidable barrier to insect dispersal. Thus, even reaching Greenland is not enough for an insect to colonise Zackenberg. Eastern Greenland, where

our work was conducted, is particularly isolated - since it isolated to the west by the inland ice, and to the SE from the nearest potential source of immigrants (Iceland) by the Denmark strait. The Denmark strait is dominated by a cold current sweeping down directly from the North Pole, transporting massive amounts of ice in the process. Finally, the coast line is deeply serrated by fjords running perpendicular to the narrow ice-free coastline. The odds of an insect species dispersing north in sufficient numbers to establish a viable population, at least under current conditions, are therefore extremely slim. This isolation means that the sets of plants and insects present at Zackenberg are highly consistent over time.

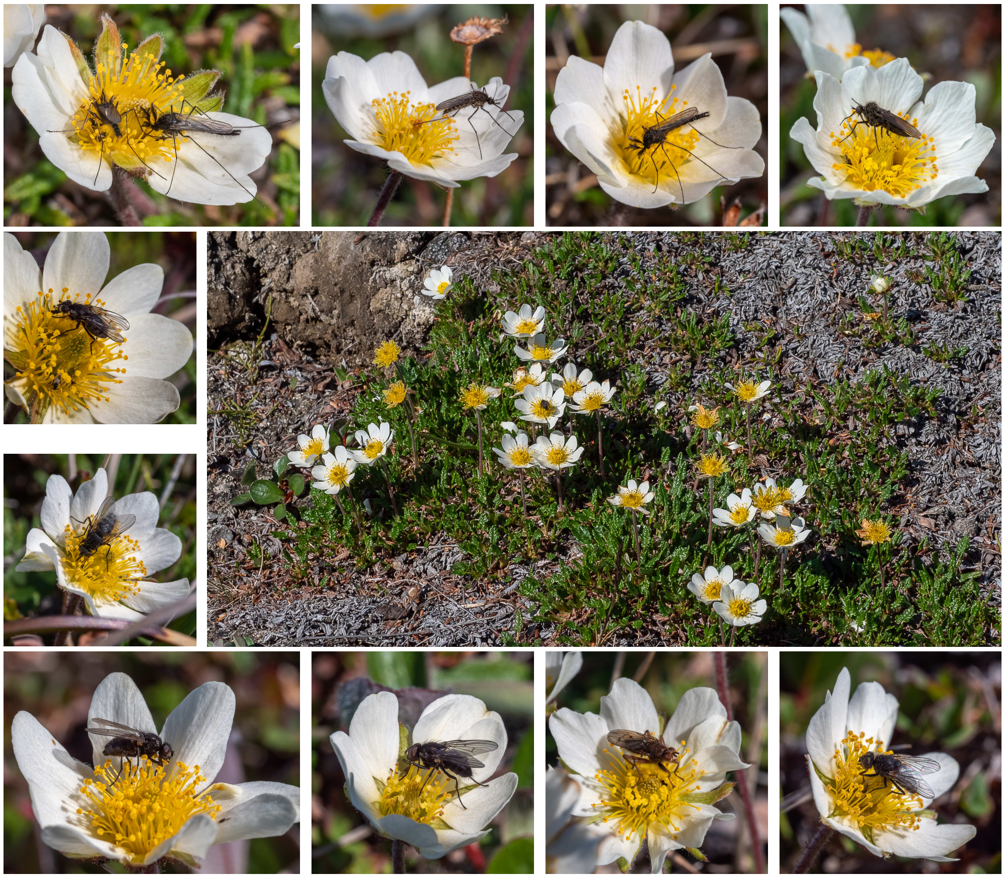

Figure S2: *Dryas* flowers are open disks with brush-like styles surrounded by anthers. Nectar is secreted under the anthers. Most flower-visitors seeking nectar will contact the anthers and be coated in pollen. Here, a variety of insects are visiting *Dryas* flowers and contacting anthers. Pollen grains are visible on the bodies of many of the insects. Photographs kindly provided by Piotr Łukasik.

Section S2 Network construction

Species in the 1996-2011 dataset were identified using morphology while the 2016 dataset primarily used DNA barcoding (see below for detailed methods). This methodological difference, together with ongoing revisions to insect taxonomies, mean that there are some taxonomic mis-matches between datasets. We resolved such mis-matches as much as possible to create one common set of taxa across all datasets. Where taxonomies are poorly resolved or morphological species identification was tentative (*Atractodes*, *Draba*, *Stenomacrus* and all Chironomidae), we grouped species to the genus level. These species are morphologically similar and likely to perform similar ecological functions. Similarly, two morphologically and ecologically similar Bombus species were grouped together as they are difficult to distinguish without DNA identification. For genera where only one species is known from Zackenberg (e.g., *Armeria*, *Colias*) all observed taxa were assumed to be the known species. Species belonging to genera not known from Zackenberg were grouped to genus. These species could be rare long-time residents that have escaped prior sampling or recent immigrants to the study site. For a full list of accepted taxon names and synonyms, see Table S1, *Appendix S3*.

Two groups of flower visitors, spiders and mites, were observed in the 2016 data but not included in the 1996-2011 data. As these groups were almost certainly present at Zackenberg at that time, including them in the 2016 dataset would artificially inflate the difference between this and earlier years. Further, sample sizes of spiders and mites captured in 2016 were very small (4 and 1, respectively) so that we cannot base strong conclusions on this sample. Finally, the pollen that was recovered from spiders and mites does not provide strong evidence that these individuals moved between plants and could be viable pollinators. The mite carried only pollen from *Salix arctica*, the plant where it was captured. Of the spiders, two also carried only pollen from the flowers where they were captured (one only 4 grains). One of the spiders with mixed pollen was collected with a scathophagid prey, meaning that we cannot clearly attribute the foreign pollen to the spider. The final spider did carry pollen from multiple flowers (*Dryas*, *Salix arctica*, *Silene acaulis*, *Arenaria*, and *Silene* sp.). However, except for the plant of capture (*Dryas*) these links were supported by only 1-4 pollen grains. As such small numbers of pollen grains could easily have been transported by insects visiting the *Dryas* flower, we do not consider this single spider sufficient justification to change network construction between 2016 and the earlier years. We therefore removed all observations for these species in 2016.

Using these datasets, we constructed two types of networks. Flower-visitor networks contained data from 1996-2016, while pollen-transport networks contained only data from 2016. The two network types each record plant-insect interactions, but the meaning of interaction weights varied between network types.

Section S2.1 DNA barcoding

The pollinators were identified in the field as accurately as possible, and when species level identification on the field was not obtained, the pollinator was identified with DNA barcoding. One or several legs (depending on the size) or the head of a pollinator was taken for the DNA extraction, removing it/ them with tweezers cleaned with ethanol (95%) and flamed, and placed into separate 1.5 ml tubes. If the individual was very small, the whole individual was used. Samples were stored in 30μl of 96% EtOH in -20°C before extraction. Salt extraction protocol

(Aljanabi & Martinez (1997) following modifications as in Vesterinen *et al.* (2016)) was used for the DNA extraction. For DNA barcoding of pollinators from plot 1 the CO1 region was amplified with the primers HCO-1490 (GGTCAACAAATCATAAAGATATTGG (Folmer *et al.*, 1994)) and LCO-2198 (TAAACTTCAGGGTGACCAAAAAATCA ((Folmer *et al.*, 1994))). The PCR reaction was done in a total volume of 15 μl, each containing 7.5 μl MyTaq Red Mix (Bioline, London, UK), 4.6 μl DNA-free water, 0.45 μl of each primer (10 μM) and 2 μl of DNA extract. PCR cycling conditions were as follows: initial denaturation for 5 min at 95 °C, 35 cycles of 40 s 95 °C, 60 s 48 °C, 30 s 72 °C, and ending with final extension for 5 min at 72 °C. For each 96 well plate two negative controls were included. All the amplicons were checked on a 1% agarose gel and imaged with a BioRad imager and when a reaction had not produced a clear band, the PCR was repeated. Ten μl MQ water was added into each sample before sending to be sequenced at Macrogen Ltd. Sequencing was done to one direction only, with the LCO-2198 as the sequencing primer. For the samples for which the amplification and/ or sequencing had failed, the PCR and sequencing were redone with the same conditions, with the exception that the sequencing was done in different direction, with the primer HCO-2198. The sequences were viewed, primer sequences were removed and low quality ends were trimmed in program Geneious (version 11, Biomatters Ltd). The sequences were then taxonomically assigned by comparison to the CO1 species database in the Barcode of Life Datasystems (BOLD, (Ratnasingham & Hebert, 2007)). Identifications with similarity of at least 98% were accepted if the species was known to occur in Greenland (based on Wirta *et al.* (2016) and all records from Greenland in BOLD, 24.4.2021). When the sequence matched to multiple species, but only to one species known from Greenland, the sequence was assigned to that. If the sequence matched to multiple species from Greenland with >98% similarity, the sample was assigned to genus. The sequences which did not find a species specific match of at least 98% similarity, were then compared to the All barcode records database in BOLD and identification was accepted for at least 98% similarity to a specimen identified to genus known from Greenland (based on Wirta *et al.* (2016)).

Section S2.2 Flower-visitor networks

Visitation was recorded between a focal insect and the plant species from which it was collected (i.e., one interaction per pollinator individual). These interactions were used to create networks where the weight of each link is the number of insect individuals visiting the focal plant taxon during either a given week (weekly networks) or year (annual networks). Where multiple individuals from the same taxon were collected, the weight for the interaction was assigned as the number of barcoded individuals. Where not all individuals of a proposed morphospecies were barcoded, only one individual per barcode was counted regardless of the number of individuals in a sample (to avoid over-representing the strength of these links in case non-barcoded individuals were of different species).

Section S2.3 Pollen-transport networks

Pollen transport was recorded between a focal insect and any plant species represented by pollen swabbed from the insect (usually giving multiple interactions per pollinator individual). These interactions were used to create networks where the weight of each link is the total number of pollen grains for a plant species divided by the number of focal pollinator individuals carrying

focal plant pollen (i.e., average number of pollen grains carried by an individual pollinator). Where samples contained multiple individuals, pollen counts were divided equally among individuals. Some samples contained individuals from multiple taxa. As it is impossible to tell which individual pollen in a shared sample came from, we included these individuals in the flower-visitor networks only.

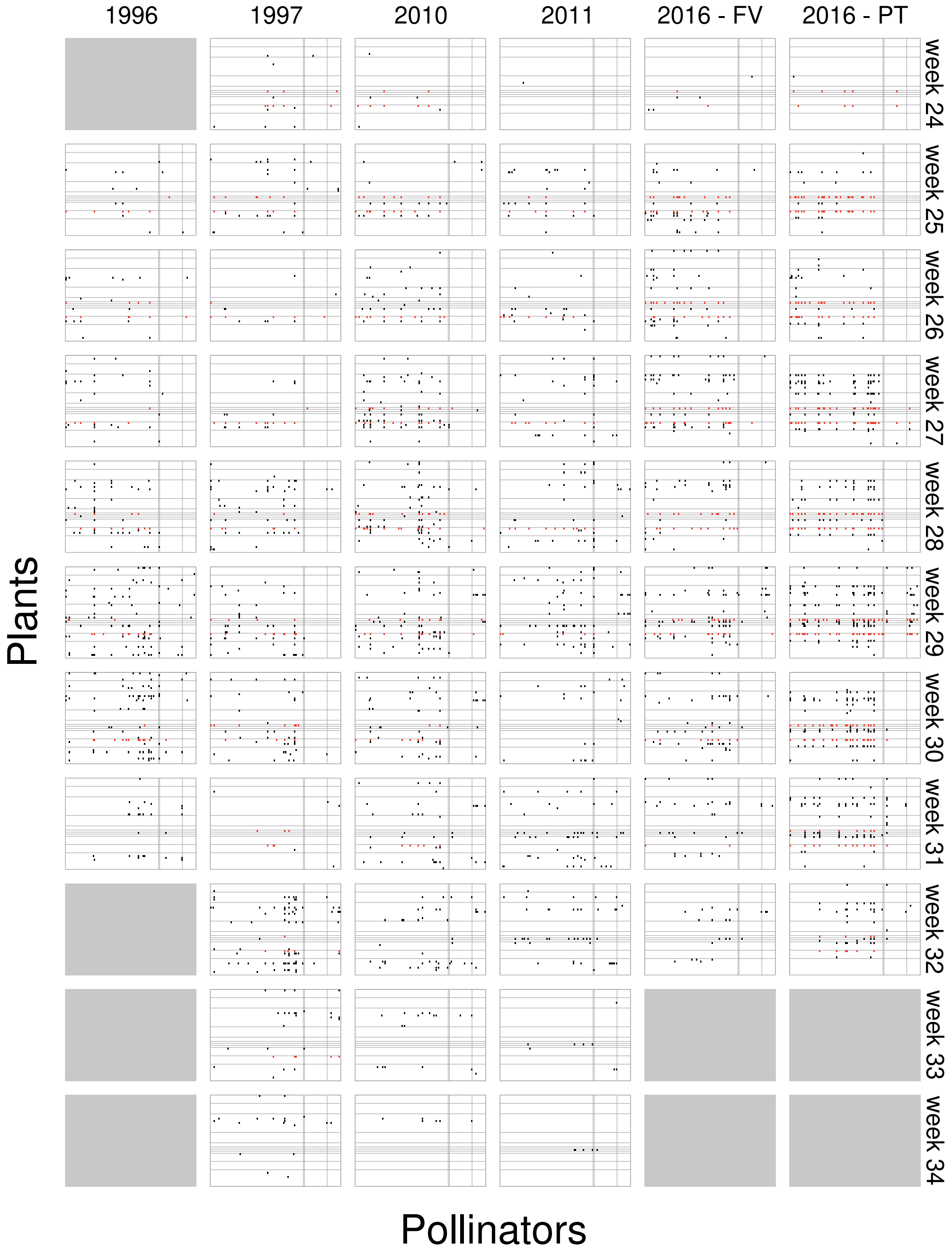

Figure S3: Only a small fraction of the interactions in the multi-year metaweb were observed in any given week. Binary weekly (weeks 24-32,) networks of flower visitor (1996, 1997, 2010, 2011, and 2016) and pollen transport (2016 only, indicated by '2016-PT') interactions. Interactions from 1996-2011 are derived from (Rasmussen *et al.*, 2013). Taxa are ordered by order, then alphabetically within order. Thin vertical and horizontal lines divide orders. Black boxes indicate an interaction. Weeks indicated by grey boxes were not included in annual sampling due to inclement weather. Two key plants, *Dryas* and *Salix arctica*, are highlighted in red as in Fig. 1 (*Main text*).

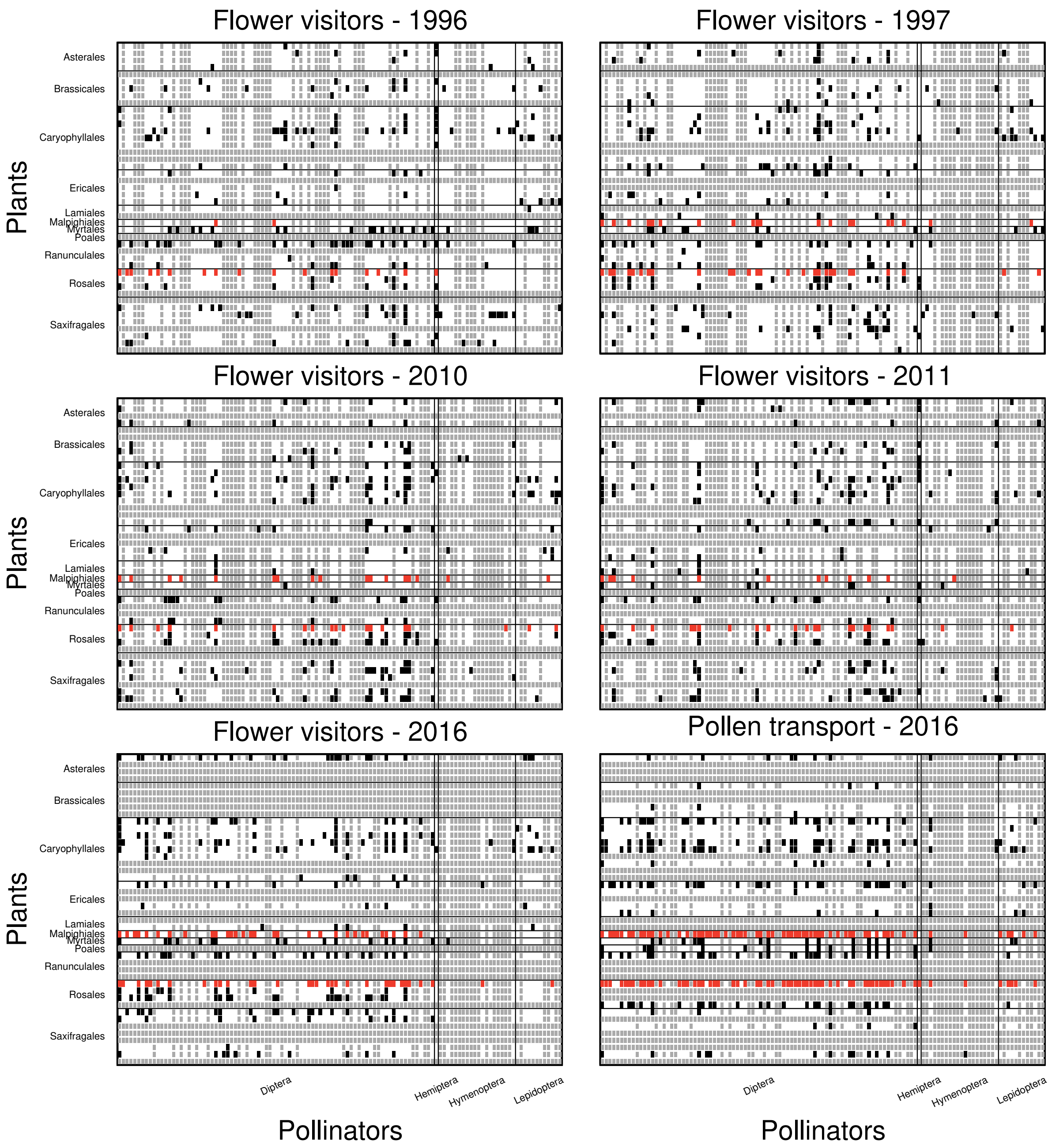

Figure S4: Binary networks of flower-visitor and pollen-transport interactions occurring in each year. Taxa are ordered alphabetically by order, then taxon within order. Thin vertical and horizontal lines divide orders. Black boxes indicate an interaction. Two key plants in the system (*Dryas*; Rosaceae, Rosales and *Salix arctica*; Salicaceae, Malpighiales) are highlighted in red. Grey boxes indicate an interaction for which the plant or insect was not included in the focal network (for example, plants with pollen collected from insects but not observed directly, or pollinators which were captured but from which pollen was not recovered). Turnover between years was extremely high for Hymenoptera, with most taxa being oberved in only one year.

Section S3 Accepted names and synonyms for all taxa

Table S1: Taxon names included in our final networks and all synonyms for each accepted name.

Group	Order	Family	Taxon	Aliases
Insect	Diptera	Agromyzidae	Phytomyza sp.	Phytomyza fuscula
Insect	Diptera	Anthomyiidae	Delia echinata	-
Insect	Diptera	Anthomyiidae	Delia platura	-
Insect	Diptera	Anthomyiidae	Egle groenlandica	Egle inermis
Insect	Diptera	Anthomyiidae	Eutrichota tunicata	-
Insect	Diptera	Anthomyiidae	Fucellia pictipennis	-
Insect	Diptera	Anthomyiidae	Paradelia arctica	-
Insect	Diptera	Anthomyiidae	Pegomya icterica	-
Insect	Diptera	Anthomyiidae	Zaphne sp.	Zaphne, Zaphne divisa, Zaphne frontata, Zaphne occidentalis, Zaphne divisa
Insect	Diptera	Calliphoridae	Cynomya mortuorum	-
Insect	Diptera	Calliphoridae	Protophormia terraenovae	-
Insect	Diptera	Ceratopogonidae	Brachypogon sp.	Brachypogon sp., Brachypogon sp. BOLD:AAG6532 voucher BIOUG01915-A06, Brachypogon sp. BOLD:AAG6532 voucher BIOUG01915-A06
Insect	Diptera	Ceratopogonidae	Culicoides	Culicoides sp., Culicoides sp.
Insect	Diptera	Ceratopogonidae	Forcipomyia sp 1	-
Insect	Diptera	Chironomidae	Allocladius	Allocladius nanseni
Insect	Diptera	Chironomidae	Chaetocladius	Chaetocladius, Chaetocladius
Insect	Diptera	Chironomidae	Chironomidae	Chironomidae
Insect	Diptera	Chironomidae	Chironomus	Chironomus hyperboreus, Chironomus pseudothummi, Chironomus sp., Chironomus sp.
Insect	Diptera	Chironomidae	Cricotopus	Cricotopus, Cricotopus magus, Cricotopus obnixus, Cricotopus patens, Cricotopus sp., Cricotopus sp. 27ES, Cricotopus sp. 27ES
Insect	Diptera	Chironomidae	Eukiefferiella	Eukiefferiella sp 1
Insect	Diptera	Chironomidae	Limnophyes	Limnophyes, Limnophyes asquamatus, Limnophyes brachytomus, Limnophyes cf. natalensis, Limnophyes cf. ninae, Limnophyes minimus, Limnophyes ninae, Limnophyes sp., Limnophyes cf. ninae
Insect	Diptera	Chironomidae	Metriocnemus	Metriocnemus, Metriocnemus brusti, Metriocnemus sp., Metriocnemus sp. 1ES, Metriocnemus sp. BOLD-2016 voucher BIOUG17157-G04, Metriocnemus sp.

Table S1 (cont.): Taxon names included in our final networks and all synonyms for each accepted name.

Group	Order	Family	Taxon	Aliases
Insect	Diptera	Chironomidae	Orthocladius	Orthocladius sp.
Insect	Diptera	Chironomidae	Paraphaenocladius	Paraphaenocladius impensus
Insect	Diptera	Chironomidae	Procladius	Procladius crassinervis, Procladius paragretis, Procladius sp, Procladius sp
Insect	Diptera	Chironomidae	Psectrocladius	Psectrocladius limbatellus
Insect	Diptera	Chironomidae	Rheocricotopus	Rheocricotopus chapmani
Insect	Diptera	Chironomidae	Smittia	Pseudosmittia cf nanseni, Pseudosmittia nanseni, Pseudosmittia sp., Smittia, Smittia edwardsi, Smittia extrema, Smittia sp., Smittia sp. BOLD-2016 voucher 08WOLVES-01289, Smittia sp. BOLD-2016 voucher BIOUG16809-C09, Smittia sp. ES12 voucher BIOUG11304-F07, Smittia sp 17, Smittia sp 2, Smittia sp aff edwardsi, Smittia sp x, Pseudosmittia cf nanseni
Insect	Diptera	Chironomidae	Tanytarsus	Tanytarsus sp 1
Insect	Diptera	Culicidae	Aedes sp.	Aedes, Aedes impiger, Aedes impiger
Insect	Diptera	Diptera	Diptera	-
Insect	Diptera	Empididae	Rhamphomyia filicauda	-
Insect	Diptera	Empididae	Rhamphomyia nigrita	Ramphomyia nigrita
Insect	Diptera	Empididae	Rhamphomyia sp.	Empididae
Insect	Diptera	Heleomyzidae	Neoleria prominens	-
Insect	Diptera	Muscidae	Drymeia groenlandica	-
Insect	Diptera	Muscidae	Drymeia segnis	-
Insect	Diptera	Muscidae	Limnophora groenlandica	-
Insect	Diptera	Muscidae	Lophosceles minimus	-
Insect	Diptera	Muscidae	Muscidae	-
Insect	Diptera	Muscidae	Phaonia bidentata	-
Insect	Diptera	Muscidae	Spilogona	-
Insect	Diptera	Muscidae	Spilogona almqvistii	-
Insect	Diptera	Muscidae	Spilogona arcticola	-
Insect	Diptera	Muscidae	Spilogona deflorata	-
Insect	Diptera	Muscidae	Spilogona denudata	-
Insect	Diptera	Muscidae	Spilogona dorsata	Spilogona imitatrix
Insect	Diptera	Muscidae	Spilogona malaisei	-
Insect	Diptera	Muscidae	Spilogona megastoma	-
Insect	Diptera	Muscidae	Spilogona melanosoma	-
Insect	Diptera	Muscidae	Spilogona micans	-

Table S1 (cont.): Taxon names included in our final networks and all synonyms for each accepted name.

Group	Order	Family	Taxon	Aliases
Insect	Diptera	Muscidae	Spilogona sanctipauli	-
Insect	Diptera	Muscidae	Spilogona sp.	Spilogona extensa, Spilogona obsoleta, Spilogona obsoleta
Insect	Diptera	Muscidae	Spilogona tendipes?	-
Insect	Diptera	Muscidae	Spilogona tornensis	-
Insect	Diptera	Muscidae	Spilogona zaitzevi	-
Insect	Diptera	Mycetophilidae	Exechia micans	-
Insect	Diptera	Mycetophilidae	Phronia egregia	-
Insect	Diptera	Phoridae	Megaselia arcticae	-
Insect	Diptera	Piophilidae	Lasiopiophila pilosa	-
Insect	Diptera	Scathophagidae	Gonarcticus arcticus	-
Insect	Diptera	Scathophagidae	Scathophaga furcata	-
Insect	Diptera	Scathophagidae	Scathophaga nigripalpis	-
Insect	Diptera	Scathophagidae	Scathophagidae	Scatophagidae
Insect	Diptera	Sciaridae	Bradysia sp.	Bradysia n. sp.
Insect	Diptera	Sciaridae	Lycoriella abbrevinervis	-
Insect	Diptera	Sciaridae	Lycoriella flavipeda	-
Insect	Diptera	Sciaridae	Lycoriella modesta	-
Insect	Diptera	Sciaridae	Lycoriella riparia	-
Insect	Diptera	Sciaridae	Lycoriella sp.	Lycoriella? sp 1
Insect	Diptera	Sciaridae	Lycoriella vitticollis	-
Insect	Diptera	Sciaridae	Scatopsciara sp 1	-
Insect	Diptera	Sciaridae	Sciaridae	-
Insect	Diptera	Syrphidae	Eupeodes punctifer	-
Insect	Diptera	Syrphidae	Eupeodes rufipunctatus	-
Insect	Diptera	Syrphidae	Eupeodes sp.	Eupeodes luniger
Insect	Diptera	Syrphidae	Helophilus groenlandicus	Helophilus groenladicus
Insect	Diptera	Syrphidae	Helophilus lapponicus	-
Insect	Diptera	Syrphidae	Parasyrphus	Parasyrphus tarsatus
Insect	Diptera	Syrphidae	Platycheirus	-
Insect	Diptera	Syrphidae	Platycheirus carinatus	-
Insect	Diptera	Syrphidae	Platycheirus groenlandicus	-
Insect	Diptera	Syrphidae	Platycheirus lundbecki	Platycheirus coerulescens1
Insect	Diptera	Syrphidae	Platycheirus sp.	Platycheirus chilosia
Insect	Diptera	Syrphidae	Syrphus torvus	-
Insect	Diptera	Tachinidae	Peleteria aenea	-

Table S1 (cont.): Taxon names included in our final networks and all synonyms for each accepted name.

Group	Order	Family	Taxon	Aliases
Insect	Diptera	Tipulidae	Nephrotoma lundbecki	-
Insect	Diptera	Tipulidae	Tipula arctica	Tipula
Insect	Hemiptera	Lygaeidae	Nysius groenlandicus	-
Insect	Hymenoptera	Apidae	Bombus	Bombus, Bombus hyperboreus, Bombus polaris, Bombus
Insect	Hymenoptera	Braconidae	Braconidae	-
Insect	Hymenoptera	Braconidae	Cotesia hallii	-
Insect	Hymenoptera	Braconidae	Dacnusa groenlandica	-
Insect	Hymenoptera	Braconidae	Microplitis lugubris	-
Insect	Hymenoptera	Braconidae	Praon brevistigma	-
Insect	Hymenoptera	Braconidae	Protapanteles fulvipes	-
Insect	Hymenoptera	Encyrtidae	Pseudencyrtus sp 1	-
Insect	Hymenoptera	Eulophidae	Aprostocetus meltoftei	-
Insect	Hymenoptera	Ichneumonidae	Atractodes	Atractodes alpestris, Atractodes aterrimus, Atractodes alpestris
Insect	Hymenoptera	Ichneumonidae	Buathra laborator	-
Insect	Hymenoptera	Ichneumonidae	Cryptinae sp.	-
Insect	Hymenoptera	Ichneumonidae	Diplazontinae sp.	-
Insect	Hymenoptera	Ichneumonidae	Gelis maesticolor	-
Insect	Hymenoptera	Ichneumonidae	Gelis sp 1	-
Insect	Hymenoptera	Ichneumonidae	Neurateles? sp 1	-
Insect	Hymenoptera	Ichneumonidae	Picrostigeus? sp 1	-
Insect	Hymenoptera	Ichneumonidae	Plectiscus	Plectiscus? sp 1, Plectiscus? sp 1
Insect	Hymenoptera	Ichneumonidae	Stenomacrus	Stenomacrus? sp 2, Stenomacrus micropennis, Stenomacrus micropennis?, Stenomacrus sp. 1ZERO, Stenomacrus sp 1, Stenomacrus sp B, Stenomacrus micropennis
Insect	Hymenoptera	Megaspilidae	Dendrocerus sp 1	-
Insect	Hymenoptera	Pteromalidae	Pachyneuron groenlandicum	-
Insect	Lepidoptera	Geometridae	Entephria kidluitata	Entephria punctipes
Insect	Lepidoptera	Lycaenidae	Agriades glandon	Plebeius glandon
Insect	Lepidoptera	Noctuidae	Apamea zeta	Apamea exulis
Insect	Lepidoptera	Noctuidae	Euxoa adumbrata	Euxoa drewseni
Insect	Lepidoptera	Noctuidae	Polia richardsoni	Pohlia
Insect	Lepidoptera	Noctuidae	Sympistis nigrita zetterstedtii	Sympistis lapponica, Sympistis nigrita, Sympistis nigrita
Insect	Lepidoptera	Noctuidae	Syngrapha parilis	-
Insect	Lepidoptera	Nymphalidae	Boloria chariclea	Boloria

Table S1 (cont.): Taxon names included in our final networks and all synonyms for each accepted name.

Group	Order	Family	Taxon	Aliases
Insect	Lepidoptera	Pieridae	Colias hecla	Colias gigantea
Insect	Lepidoptera	Tortricidae	Argyroploce aquilonana	Argyroploce aquilonana
Insect	Lepidoptera	Tortricidae	Olethreutes inquitana	-
Insect	Lepidoptera	Tortricidae	Olethreutes mengelana	-

Table S1 (cont.): Taxon names included in our final networks and all synonyms for each accepted name.

Group	Order	Family	Taxon	Aliases
Plant	Asterales	Asteraceae	Arnica angustifolia	-
Plant	Asterales	Asteraceae	Erigeron compositus	-
Plant	Asterales	Asteraceae	Taraxacum arcticum	-
Plant	Asterales	Asteraceae	Taraxacum phymatocarpum	-
Plant	Asterales	Campanulaceae	Campanula rotundifolia subsp. gieseckiana	Campanula giesekiana
Plant	Brassicales	Brassicaceae	Braya glabella	Braya, Braya glabella
Plant	Brassicales	Brassicaceae	Cardamine pratensis	Cardamine pratensis
Plant	Brassicales	Brassicaceae	Cochlearia groenlandica	-
Plant	Brassicales	Brassicaceae	Draba sp	Draba arctica, Draba lactea, Draba sp.
Plant	Brassicales	Brassicaceae	Lesquerella arctica	Lesquerella
Plant	Caryophyllales	Caryophyllaceae	Arenaria pseudofrigida	Arenaria
Plant	Caryophyllales	Caryophyllaceae	Cerastium arcticum	Cerastium
Plant	Caryophyllales	Caryophyllaceae	Silene acaulis	-
Plant	Caryophyllales	Caryophyllaceae	Silene sorensenis	Melandrium triflorum
Plant	Caryophyllales	Caryophyllaceae	Silene sp.	-
Plant	Caryophyllales	Caryophyllaceae	Stellaria humifusa	-
Plant	Caryophyllales	Caryophyllaceae	Stellaria longipes	-
Plant	Caryophyllales	Plumbaginaceae	Armeria scabra	Armeria
Plant	Caryophyllales	Polygonaceae	Bistorta vivipara	Polygonum viviparum
Plant	Ericales	Ericaceae	Cassiope tetragona	-
Plant	Ericales	Ericaceae	Pyrola grandiflora	-
Plant	Ericales	Ericaceae	Rhododendron lapponicum	Rhododentron
Plant	Ericales	Ericaceae	Vaccinium uliginosum	-
Plant	Ericales	Polemoniaceae	Polemonium boreale	-
Plant	Lamiales	Orobanchaceae	Pedicularis flammea	-
Plant	Lamiales	Orobanchaceae	Pedicularis hirsuta	-
Plant	Malpighiales	Salicaceae	Salix arctica	-
Plant	Myrtales	Onagraceae	Chamaenerion latifolium	Chamerion latifolium, Chamerlion latifolium
Plant	Poales	Poaceae	Deschampsia sp.	Descampsia
Plant	Ranunculales	Papaveraceae	Papaver radicatum	-
Plant	Ranunculales	Ranunculaceae	Ranunculus hyperboreus	-
Plant	Ranunculales	Ranunculaceae	Ranunculus pygmaeus	-
Plant	Ranunculales	Ranunculaceae	Ranunculus sulphureus	-

Table S1 (cont.): Taxon names included in our final networks and all synonyms for each accepted name.

Group	Order	Family	Taxon	Aliases
Plant	Rosales	Rosaceae	Dryas octopetala	Dryas integrifoliaXoctopetala
Plant	Rosales	Rosaceae	Potentilla hyparctica	Potentialla hypartica
Plant	Rosales	Rosaceae	Potentilla rubricaulis	Potentillia rubricaulis
Plant	Rosales	Rosaceae	Potentilla sp.	-
Plant	Saxifragales	Saxifragaceae	Micranthes nivalis	-
Plant	Saxifragales	Saxifragaceae	Saxifraga cernua	-
Plant	Saxifragales	Saxifragaceae	Saxifraga cespitosa	Saxifraga caespitosa, Saxifraga caespitosa?
Plant	Saxifragales	Saxifragaceae	Saxifraga hirculus	-
Plant	Saxifragales	Saxifragaceae	Saxifraga hyperborea	-
Plant	Saxifragales	Saxifragaceae	Saxifraga nivalis	-
Plant	Saxifragales	Saxifragaceae	Saxifraga oppositifolia	-
Plant	Saxifragales	Saxifragaceae	Saxifraga rivularis	-

Section S4 Pollen identification

To identify the pollen morphologically, a pollen reference collection for all Zackenberg flowering plants was created by collecting pollen from the flowering plants and imaging them with a plate scanner. Pollen was collected from each insect (after removing pollen baskets on bumble bees; no other local pollinators have pollen baskets). The insect was immersed in 1ml of a 1% sodium dodecyl sulphate (SDS) and 2% poly vinyl pyrrolidinone (PVP) solution in ultrapure water. The tube was shaken vigorously by hand for 1 minute, and then centrifuged briefly to ensure that the sample was fully immersed in the liquid. The sample was left to stand at room temperature for 5 minutes and then shaken vigorously by hand for 20 seconds. The insect was then removed to a clean 1.5ml Eppendorf tube using clean forceps and frozen at -20C for subsequent species identification (when necessary). The tube containing the detergent and pollen was centrifuged at 13000 rpm for 5 minutes before discarding the supernatant.

All the pollen that was collected from the pollinators was imaged, and the pollen grains were identified from the images. The samples were first placed onto imaging plates (Cell Carrier Ultra 96 plate, Perkin Elmer Ltd) for imaging. The original volume of the samples varied approximately 2050 μl, but for the imaging the volume was evened to 100 μl with MQ water. The pollen was imaged with the plate scanner ImageXpress Nano (Molecular Devices, US), taking 25 images per well. Green (GFP) and red (Texas red) wavelengths, an objective of 10x and exposure time of 450 ms were used. The images were viewed with the program Fiji ImageJ (Schindelin *et al.*, 2012) or IfranView (Skiljan, 2021). From each sample 10-15 images (40-60% of the sample) were counted. Note that, in some cases, pollen was not recovered from a captured insect (e.g., if the insect was captured before contacting the stigma or if pollen was lost during handling) or the recovered pollen could not be identified.

The pollinators from the single visits to *Dryas* flowers were identified by DNA metabarcoding of the CO1 region, with the primers including the linker tag tagF_LCO (tcgtcggcagcgtcagatgtgtataagagacagGGTCAACAAATCATAAAGATATTGG (Folmer *et al.*, 1994)) and tagR_CR (gtctcgtgggctcggagatgtgtataagagacagGGIGGRTAIACIGTTCA ICC (Shokralla *et al.*, 2015)). The first amplifications were done as above for the samples from plot 1 (used to construct the 2016 plant-pollinator networks). We then performed a second PCR to attach Illuminaspecific adapters and the unique dualindex combinations for each sample (Vesterinen *et al.*, 2018). It was done in a total volume of 10 μl, each containing 5 μl MyTaq Red Mix (Bioline, London, UK), 1.2 μl of each primer (2.5 μM) and 2.6 μl of the locus-specific 1st PCR product. The PCR cycling conditions were: 4 min 95 °C, 15 cycles of 20 s 98 °C, 15 s 60 °C, 30 s 72 °C, and ending with 3 min 72 °C. DNA libraries were pooled per gene region and purified using a SPRI bead protocol (Vesterinen *et al.*, 2016). The DNA concentration of the cleaned pools were measured with Qubit 2.0 (dsHS DNA Kit, ThermoFisher Scientific). The samples were sequenced on a MiSeq sequencing run with v3 chemistry with 300 cycles and 2 x 300 bp paired-end read length. Bioinformatics was done as in Vesterinen *et al.* (2018). The taxonomic assignment was done by comparison to the database of CO1 sequences from Greenland from BOLD, with the same similarity thresholds as for the sequences for samples from plot 1.

Table S2: Numbers of each taxon for which identifiable pollen was recovered. Taxa marked with '*' were removed from analyses for consistency with earlier data.

Order	Family	Genus	Species	N
Araneae*	Thomisidae	Xysticus	Xysticus deichmenni	1
Araneae*	Thomisidae	Xysticus	Xysticus labradorensis	6
Diptera	Anthomyiidae	Delia	Delia echinata	35
Diptera	Anthomyiidae	Delia	Delia platura	4
Diptera	Anthomyiidae	Eutrichota	Eutrichota tunicata	1
Diptera	Anthomyiidae	Zaphne	Zaphne sp.	10
Diptera	Calliphoridae	Protophormia	Protophormia terraenovae	8
Diptera	Ceratopogonidae	Brachypogon	Brachypogon sp.	4
Diptera	Ceratopogonidae	Culicoides	Culicoides sp.	4
Diptera	Chironomidae	Allocladius	Allocladius sp.	6
Diptera	Chironomidae	Chaetocladius	Chaetocladius sp.	10
Diptera	Chironomidae	Chironomidae	Chironomidae sp.	26
Diptera	Chironomidae	Chironomus	Chironomus sp.	2
Diptera	Chironomidae	Cricotopus	Cricotopus sp.	117
Diptera	Chironomidae	Limnophyes	Limnophyes sp.	293
Diptera	Chironomidae	Metriocnemus	Metriocnemus sp.	3
Diptera	Chironomidae	Paraphaenocladius	Paraphaenocladius sp.	6
Diptera	Chironomidae	Procladius	Procladius sp.	5
Diptera	Chironomidae	Psectrocladius	Psectrocladius sp.	15
Diptera	Chironomidae	Smittia	Smittia sp.	310
Diptera	Culicidae	Aedes	Aedes sp.	96
Diptera	Empididae	Rhamphomyia	Rhamphomyia filicauda	45
Diptera	Empididae	Rhamphomyia	Rhamphomyia nigrita	89
Diptera	Empididae	Rhamphomyia	Rhamphomyia sp.	5
Diptera	Heleomyzidae	Neoleria	Neoleria prominens	1
Diptera	Muscidae	Drymeia	Drymeia groenlandica	17
Diptera	Muscidae	Drymeia	Drymeia segnis	94
Diptera	Muscidae	Limnophora	Limnophora groenlandica	5
Diptera	Muscidae	Lophosceles	Lophosceles minimus	45
Diptera	Muscidae	Muscidae	Muscidae sp.	19
Diptera	Muscidae	Phaonia	Phaonia bidentata	2
Diptera	Muscidae	Spilogona	Spilogona almqvistii	24
Diptera	Muscidae	Spilogona	Spilogona denudata	5
Diptera	Muscidae	Spilogona	Spilogona dorsata	122
Diptera	Muscidae	Spilogona	Spilogona malaisei	2
Diptera	Muscidae	Spilogona	Spilogona megastoma	91
Diptera	Muscidae	Spilogona	Spilogona melanosoma	2
Diptera	Muscidae	Spilogona	Spilogona micans	5
Diptera	Muscidae	Spilogona	Spilogona sanctipauli	202
Diptera	Muscidae	Spilogona	Spilogona sp.	12
Diptera	Muscidae	Spilogona	Spilogona zaitzevi	13
Diptera	Scathophagidae	Gonarcticus	Gonarcticus arcticus	3
Diptera	Scathophagidae	Scathophaga	Scathophaga furcata	95
Diptera	Scathophagidae	Scathophagidae	Scathophagidae sp.	2
Diptera	Sciaridae	Bradysia	Bradysia sp.	2
Diptera	Sciaridae	Lycoriella	Lycoriella abbrevinervis	2
Diptera	Sciaridae	Lycoriella	Lycoriella flavipeda	118
Diptera	Sciaridae	Lycoriella	Lycoriella modesta	19
Diptera	Sciaridae	Lycoriella	Lycoriella riparia	10
Diptera	Sciaridae	Lycoriella	Lycoriella vitticollis	9
Diptera	Sciaridae	Sciaridae	Sciaridae sp.	22
Diptera	Syrphidae	Eupeodes	Eupeodes punctifer	4
Diptera	Syrphidae	Helophilus	Helophilus groenlandicus	11
Diptera	Syrphidae	Helophilus	Helophilus lapponicus	4
Diptera	Syrphidae	Parasyrphus	Parasyrphus sp.	38
Diptera	Syrphidae	Platycheirus	Platycheirus sp.	4
Diptera	Syrphidae	Platycheirus	Platycheirus carinatus	2
Diptera	Syrphidae	Platycheirus	Platycheirus groenlandicus	19
Diptera	Syrphidae	Platycheirus	Platycheirus lundbecki	21
Diptera	Syrphidae	Platycheirus	Platycheirus sp.	2
Diptera	Tachinidae	Peleteria	Peleteria aenea	24
Diptera	Tipulidae	Nephrotoma	Nephrotoma lundbecki	3
Hemiptera	Lygaeidae	Nysius	Nysius groenlandicus	1
Hymenoptera	Apidae	Bombus	Bombus sp.	34
Hymenoptera	Braconidae	Braconidae	Braconidae sp.	3
Hymenoptera	Braconidae	Microplitis	Microplitis lugubris	8
Hymenoptera	Ichneumonidae	Stenomacrus	Stenomacrus sp.	3
Lepidoptera	Lycaenidae	Agriades	Agriades glandon	4
Lepidoptera	Noctuidae	Euxoa	Euxoa adumbrata	4
Lepidoptera	Noctuidae	Polia	Polia richardsoni	4
Lepidoptera	Nymphalidae	Boloria	Boloria chariclea	13
Lepidoptera	Pieridae	Colias	Colias hecla	8
Lepidoptera	Tortricidae	Argyroploce	Argyroploce aquilonana	6
Mesostigmata*	Parasitidae	Parasitidae	Parasitidae sp.	1

Section S5   Estimating pollen transport

As a rough estimate of the amount of pollen from each plant species j transported by each insect taxon i each week w, we multiply the number of observed visits (n_{iw}) from each insect taxon i during week w by the mean number of pollen grains ($g_{\bar{ijw}}$) of plant j carried by individuals of insect taxon i in the 2016 pollen-transport network during week w:

$$T_{ijw} \approx n_i w \times g_{\bar{ijw}}. \tag{1}$$

To estimate the total amount of pollen transported per week, we sum T_{ijw} across all insects i. Pollen transport was variable throughout the year for both insects (Fig. S5) and plants (Fig. S6), but tended to be greatest early in the flowering season. Annual totals of pollen transport also varied widely, especially among the Diptera (Fig. S7).

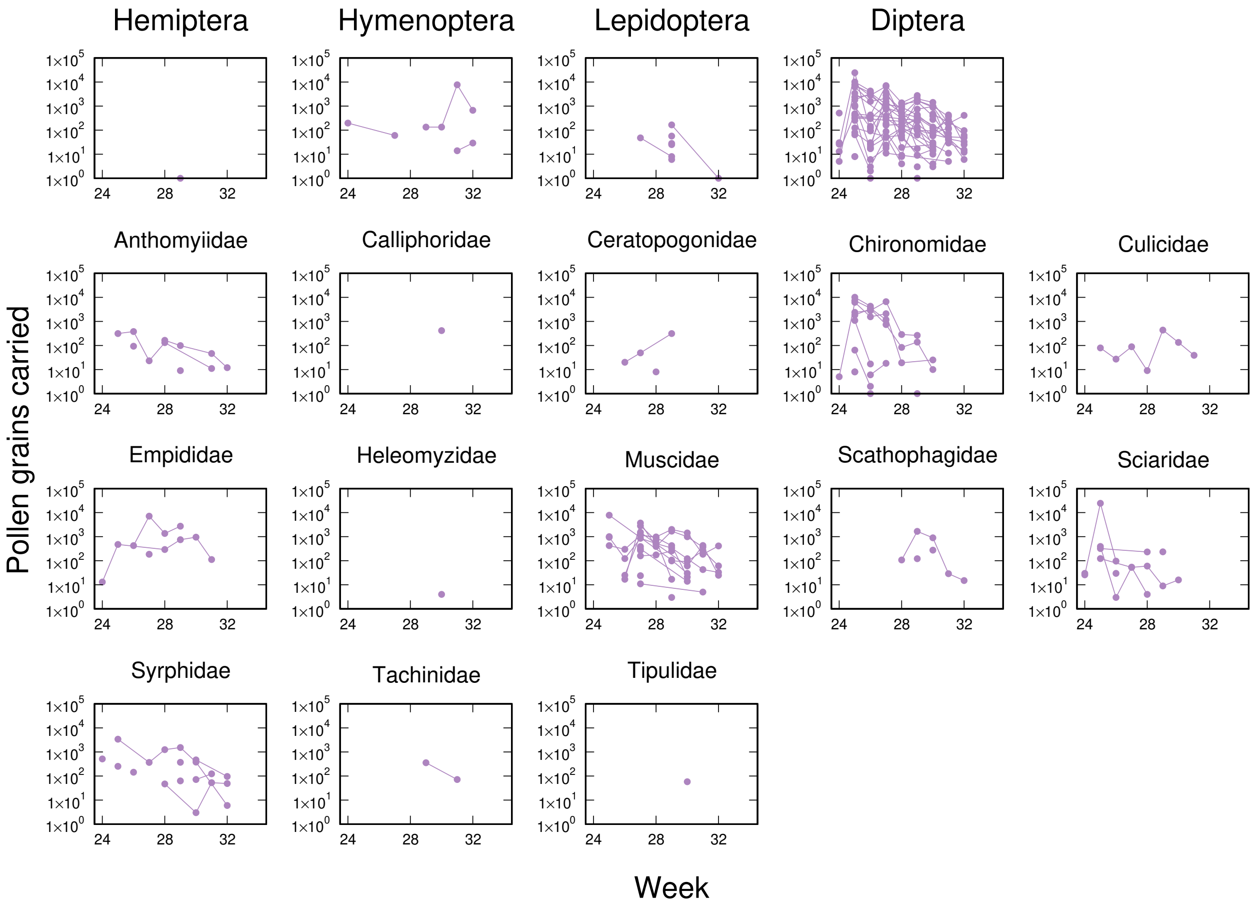

Figure S5: Total pollen carried per week by each insect species in the 2016 pollen-transport data. Insects are grouped by order; families within Diptera are shown separately.

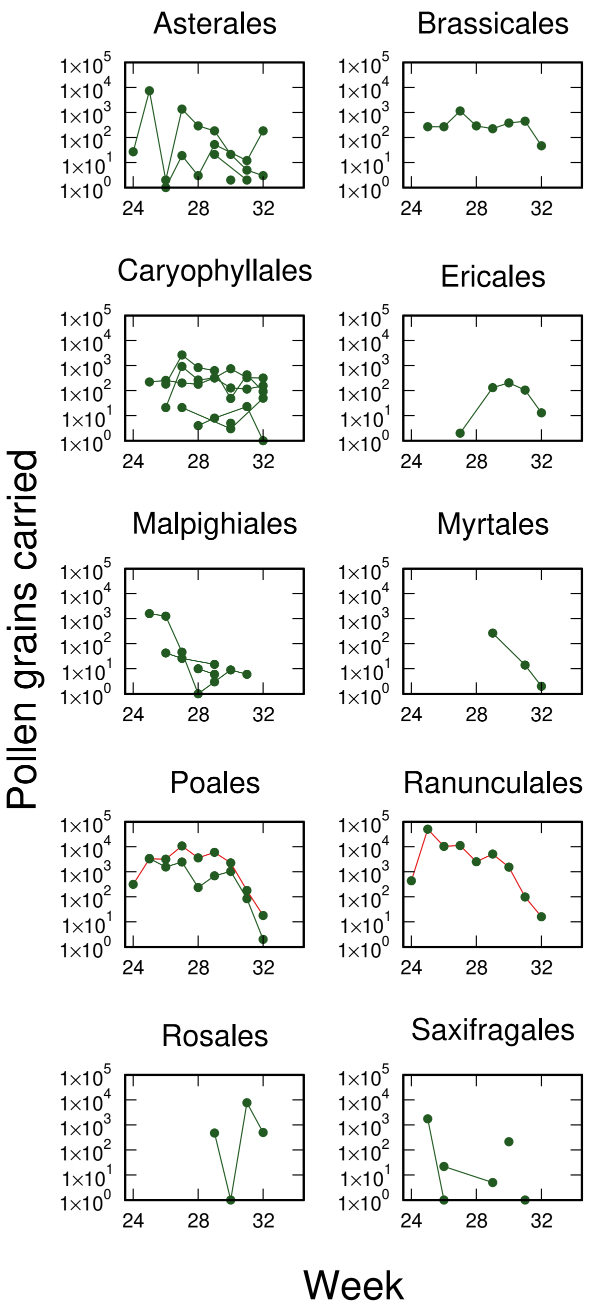

Figure S6: Total pollen from each plant species carried per week in the 2016 pollen-transport data. Plants are grouped by order.

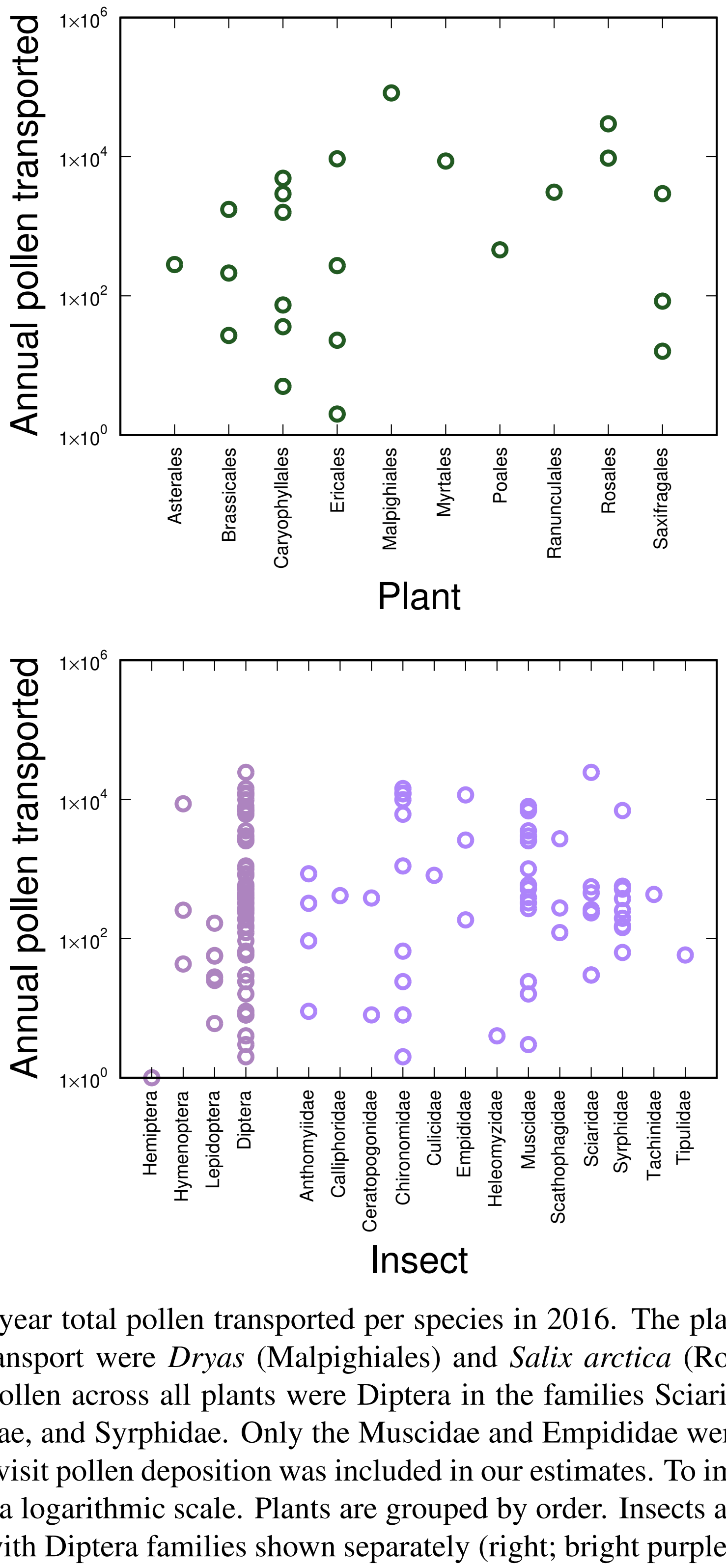

Figure S7: Whole-year total pollen transported per species in 2016. The plants with the highest estimated pollen transport were *Dryas* (Malpighiales) and *Salix arctica* (Rosales). The insects transporting most pollen across all plants were Diptera in the families Sciaridae, Chironomidae, Empididae, Muscidae, and Syrphidae. Only the Muscidae and Empididae were key pollinators of *Dryas* when single-visit pollen deposition was included in our estimates. To improve visibility, the y-axis is plotted on a logarithmic scale. Plants are grouped by order. Insects are grouped by order (left; dark purple) with Diptera families shown separately (right; bright purple).

Section S6 Estimating pollen deposition

To examine the plant-pollinator network based on pollen transferred and deposited by pollinators, the pollen deposited on a flower in a single visit was identified and counted. For this virgin *Dryas* flowers were used in plot 1 in the early season and later in the season in plot 2. *Dryas* flowers were protected by a piece of fine cloth before the flower opened, and the protective cloth was removed when the observation was initiated. Once a pollinator visited a flower, both the pollinator and all stigmas of the visited *Dryas* flower were collected separately. To identify both the con- and heterospecific pollen grains left by the visitor onto the stigma of single *Dryas* flowers, the stigma was collected separately and placed on a microscope slide, onto gel stained with fuchsin to make the pollen visible and stable. The slides were then examined under a microscope to identify the pollen, and the pollen was identified based on comparison to the local pollen reference collection.

Table S3: Mean and standard deviation (SD) of pollen grains deposited in a single visit (SVD) by insects of different families, as well as the number of individuals of each family observed (N).

Family	Mean SVD	SD SVD	N
Empididae	280.50	279.58	22
Scathophagidae	607.08	1041.31	13
Hymenoptera	998.00	742.00	2
Anthomyiidae	0.00	0.00	1
Pieridae	5312.00	0.00	1
Tachinidae	42.00	37.00	2
Muscidae	356.67	840.30	101
Chironomidae	221.91	456.32	11
Culicidae	251.67	519.58	27
Syrphidae	40.40	47.00	5

Table S4: Mean and standard error (SE) of pollen grains deposited in a single visit (SVD) by insects of different genera within families, as well as the mean and standard error of observed visits made by that genus to *Dryas* during the compilation of the 2016 flower-visitor network.

Family	Taxon	Mean SVD	SE SVD	Mean visits	SE visits
Anthomyiidae	Delia	0.0	0.0	1.75	0.415
Chironomidae	Limnophyes	11.3	3.87	18.8	6.64
Culicidae	Aedes	172	133	8.60	2.89
Empididae	Rhamphomyia	148	65.0	22.4	4.94
Muscidae	Drymeia	212	41.9	12.4	2.92
Muscidae	Spilogona	194	129	25.8	4.15
Scathophagidae	Scathophaga	774	345	1.60	0.921
Syrphidae	Helophilus	4.00	0.0	0.667	0.272
Syrphidae	Parasyrphus	63.0	0.0	1.60	0.921
Syrphidae	Platycheirus	123	0.0	6.00	1.67
Tachinidae	Peleteria	42.0	16.5	0.200	0.179

Section S7 Annual networks vs. metaweb

The annual networks were not random samples of the multi-year metaweb. In 2016, the network structures produced by the flower-visitor and pollen-transport data were significantly correlated.

Table S5: Structural properties of annual networks. Both flower-visitor (FV) and pollen-transport (PT) networks were compared to the same metaweb. Properties which were significantly different from the metaweb (i.e., either greater or lesser than 95% of the simulated networks) are highlighted in **bold** and whether these values are greater (+) or lesser (-) than the metaweb is given in parentheses. Numbers of plants and pollinators were held constant between observed and null networks.

Year	Type	Plants	Poll.s	Links	C	wC	NODF	wNODF
1996	FV	30	53	244	**0.153** (-)	**0.110** (+)	**30.6** (-)	**12.4** (-)
1997	FV	30	58	**250** (-)	**0.144** (-)	**0.107** (+)	**30.4** (-)	**11.7** (-)
2010	FV	34	67	**279** (-)	**0.122** (-)	**0.0869** (+)	**26.8** (-)	**13.4** (-)
2011	FV	32	71	**224** (-)	**0.0986** (-)	0.0822	**25.5** (-)	**10.9** (-)
2016	FV	25	83	341	**0.164** (-)	0.0802	**40.7** (-)	24.7
2016	PT	23	70	**389** (+)	0.242	0.0747	**74.7** (+)	**43.4** (+)

Table S6: Structural properties of annual flower-visitor networks. Properties which were significantly different from the metaweb (i.e., either greater or lesser than 95% of the simulated networks) are highlighted in **bold** and whether these values are greater (+) or lesser (-) than the metaweb is given in parentheses. Numbers of plants and pollinators were held constant between observed and null networks.

Year	Plants	Poll.s	Links	C	wC	NODF	wNODF
1996	30	53	244	**0.153** (-)	0.110	**30.6** (-)	**12.4** (-)
1997	30	58	250	**0.144** (-)	0.107	**30.4** (-)	**11.7** (-)
2010	34	67	**279** (-)	**0.122** (-)	0.0869	**26.8** (-)	**13.4** (-)
2011	32	71	**224** (-)	**0.0986** (-)	0.0822	**25.5** (-)	**10.9** (-)
2016	25	83	341	**0.164** (-)	**0.0802** (-)	40.7	24.7

Table S7: Despite their superficial differences, the structure of weekly networks based on flower-visitor and pollen-transport data were significantly correlated. Property values were more often significantly correlated than ranks of these values. We give the Pearson and Spearman (rank-based) correlations (ρ) for each network property shown in Fig. 3, *Main Text*, as well as the p-values for each correlation. Df=7 in all cases.

	Pearson		Spearman	
Property	ρ	p-value	ρ	p-value
Plants	2.24	0.060	63.0	0.197
Insects	15.0	$<$0.001	2.02	$<$0.001
Links	4.29	0.004	30.0	0.025
Connectance	2.03	0.082	80.0	0.385
NODF	4.24	0.004	22.0	0.011
Modularity	-0.321	0.758	102	0.708

Section S8 Weekly vs. annual networks

Table S8: Structural properties of weekly flower-visitor networks compared to same-sized null networks drawn from the corresponding annual network. Properties which were significantly different from the network (i.e., either greater or lesser than 95% of the simulated network) are highlighted in **bold**. Whether these values are greater (+) or lesser (-) than the simulated network is given in parentheses.

Year	Week	Plants	Polls	Links	C	wC	NODF	wNODF	Modularity
1996	25	9.0(0)	12.0(0)	**18.0(-)**	**0.167(-)**	**0.113(-)**	**17.0(-)**	**4.74(-)**	**0.542(+)**
1996	26	9.0(0)	16.0(0)	**27.0(-)**	**0.188(-)**	**0.112(-)**	**20.0(-)**	**1.92(-)**	0.255(0)
1996	27	14.0(0)	13.0(0)	**32.0(-)**	**0.176(-)**	0.128(0)	26.3(0)	**6.69(-)**	0.223(0)
1996	28	17.0(0)	18.0(0)	51.0(0)	0.167(0)	0.128(0)	38.2(0)	16.8(0)	0.252(0)
1996	29	24.0(0)	34.0(0)	**94.0(-)**	**0.115(-)**	**0.0801(-)**	**18.6(-)**	**5.91(-)**	**0.335(+)**
1996	30	22.0(0)	30.0(0)	**87.0(-)**	**0.132(-)**	**0.0943(-)**	**25.2(-)**	**6.11(-)**	0.272(0)
1996	31	10.0(0)	11.0(0)	26.0(0)	0.236(0)	0.151(0)	35.1(0)	13.0(0)	0.373(0)
1997	24	8.0(0)	9.0(0)	17.0(0)	0.236(0)	0.141(0)	31.0(0)	16.4(0)	0.365(0)
1997	25	13.0(0)	17.0(0)	**36.0(-)**	**0.163(-)**	**0.108(-)**	23.7(0)	9.93(0)	0.289(0)
1997	26	6.0(0)	10.0(0)	14.0(0)	0.233(0)	0.154(0)	24.6(0)	8.75(0)	0.423(0)
1997	27	6.0(0)	12.0(0)	21.0(0)	0.292(0)	0.221(0)	36.4(0)	14.2(0)	0.291(0)
1997	28	16.0(0)	22.0(0)	54.0(0)	0.153(0)	**0.0801(-)**	**22.8(-)**	7.24(0)	**0.109(-)**
1997	29	18.0(0)	22.0(0)	**56.0(-)**	**0.141(-)**	0.113(0)	26.6(0)	9.35(0)	**0.333(+)**
1997	30	18.0(0)	23.0(0)	**47.0(-)**	**0.114(-)**	**0.0829(-)**	**17.1(-)**	**5.69(-)**	**0.385(+)**
1997	31	7.0(0)	11.0(0)	**11.0(-)**	**0.143(-)**	**0.0840(-)**	**0.0(-)**	**0.0(-)**	**0.865(+)**
1997	32	20.0(0)	33.0(0)	**81.0(-)**	**0.123(-)**	**0.103(-)**	30.1(0)	10.6(0)	**0.364(+)**
1997	33	12.0(0)	14.0(0)	**28.0(-)**	**0.167(-)**	**0.107(-)**	**21.3(-)**	**2.55(-)**	0.277(0)
1997	34	9.0(0)	16.0(0)	**18.0(-)**	**0.125(-)**	**0.0743(-)**	**3.85(-)**	**2.56(-)**	**0.827(+)**
2010	24	6.0(0)	9.0(0)	12.0(0)	0.222(0)	0.131(0)	16.7(0)	4.90(0)	0.271(0)
2010	25	7.0(0)	17.0(0)	34.0(0)	0.286(0)	0.129(0)	38.1(0)	11.6(0)	0.304(0)
2010	26	12.0(0)	23.0(0)	**39.0(-)**	**0.141(-)**	0.117(0)	22.5(0)	12.2(0)	0.332(0)
2010	27	19.0(0)	26.0(0)	76.0(0)	0.154(0)	0.119(0)	32.0(0)	17.9(0)	0.274(0)
2010	28	23.0(0)	31.0(0)	**83.0(-)**	**0.116(-)**	0.0975(0)	26.1(0)	13.8(0)	**0.381(+)**
2010	29	22.0(0)	35.0(0)	**79.0(-)**	**0.103(-)**	**0.0847(-)**	**20.6(-)**	**8.18(-)**	**0.440(+)**
2010	30	18.0(0)	26.0(0)	**55.0(-)**	**0.118(-)**	**0.0766(-)**	**14.8(-)**	**5.38(-)**	**0.439(+)**
2010	31	14.0(0)	26.0(0)	**42.0(-)**	**0.115(-)**	**0.0736(-)**	**14.1(-)**	**6.13(-)**	**0.507(+)**
2010	32	14.0(0)	23.0(0)	**42.0(-)**	**0.130(-)**	**0.0843(-)**	**18.1(-)**	**5.14(-)**	**0.453(+)**
2010	33	6.0(0)	14.0(0)	**17.0(-)**	**0.202(-)**	0.128(0)	**12.1(-)**	**0.943(-)**	**0.623(+)**
2010	34	2.0(0)	5.0(0)	6.0(0)	0.600(0)	0.290(0)	40.9(0)	9.09(0)	0.374(0)
2011	24	NA	NA	NA	NA	NA	NA	NA	NA
2011	25	9.0(0)	17.0(0)	31.0(0)	0.203(0)	0.120(0)	28.4(0)	7.46(0)	0.399(0)
2011	26	11.0(0)	17.0(0)	**25.0(-)**	**0.134(-)**	**0.0929(-)**	**11.0(-)**	**4.84(-)**	**0.596(+)**
2011	27	14.0(0)	17.0(0)	36.0(0)	0.151(0)	0.123(0)	29.2(0)	18.3(0)	0.406(0)
2011	28	17.0(0)	25.0(0)	**46.0(-)**	**0.108(-)**	**0.0892(-)**	21.2(0)	10.7(0)	0.290(0)
2011	29	22.0(0)	25.0(0)	70.0(0)	0.127(0)	0.106(0)	28.0(0)	10.5(0)	0.314(0)
2011	30	11.0(0)	15.0(0)	**22.0(-)**	**0.133(-)**	**0.0954(-)**	**10.9(-)**	**4.38(-)**	**0.641(+)**
2011	31	12.0(0)	32.0(0)	**49.0(-)**	**0.128(-)**	**0.0911(-)**	**16.1(-)**	**4.92(-)**	0.518(0)
2011	32	9.0(0)	31.0(0)	**43.0(-)**	**0.154(-)**	0.105(0)	19.3(0)	7.53(0)	0.515(0)
2011	33	4.0(0)	7.0(0)	8.0(0)	0.286(0)	0.179(0)	7.41(0)	7.41(0)	**0.706(+)**
2011	34	NA	NA	NA	NA	NA	NA	NA	NA
2016	24	5.0(0)	6.0(0)	15.0(0)	0.5(0)	0.267(0)	**43.0(-)**	**0.0(-)**	0.182(0)
2016	25	14.0(0)	31.0(0)	**97.0(-)**	**0.224(-)**	0.126(0)	**59.1(-)**	**24.5(-)**	**0.255(+)**
2016	26	18.0(0)	35.0(0)	**107.0(-)**	**0.170(-)**	0.105(0)	**51.6(-)**	**27.8(-)**	0.232(0)
2016	27	21.0(0)	36.0(0)	173.0(0)	0.229(0)	0.139(0)	60.5(0)	28.2(0)	0.218(0)
2016	28	17.0(0)	34.0(0)	140.0(0)	0.242(0)	0.141(0)	62.0(0)	**28.1(-)**	0.240(0)
2016	29	23.0(0)	38.0(0)	202.0(0)	0.231(0)	**0.178(+)**	62.8(0)	**19.8(-)**	0.160(0)
2016	30	22.0(0)	38.0(0)	**168.0(-)**	**0.201(-)**	**0.149(+)**	**56.9(-)**	**15.9(-)**	**0.293(+)**
2016	31	21.0(0)	28.0(0)	**122.0(-)**	**0.207(-)**	0.132(0)	**46.7(-)**	**10.8(-)**	**0.276(+)**
2016	32	18.0(0)	15.0(0)	**59.0(-)**	**0.219(-)**	0.148(0)	**34.1(-)**	**6.65(-)**	**0.250(+)**

Section S9 Activity periods

Section S9.1 Methods

We measured the activity period of each plant and insect taxon in each year that it was observed. Because the first and last open flower or insect visit can easily be missed, we record active periods as the range of weeks in which flowers were visited or insects observed visiting plants rather than the range of days. Specifically, we recorded the number of insects observed or flowers visited in each week, in each of the flower-visitor networks. We did not include data from the pollen-transport networks as we have no information on how many flowers contributed to an insect's pollen load.

We then tested whether the set of activity periods are consistently related to the year (e.g., warm years or cold years), taxonomic orders, or their interactions using two PERMANOVAs (one for plants and one for insects). We fit each PERMANOVA using the R (R Core Team, 2016) function 'adonis' from the package *vegan* (Oksanen *et al.*, 2019), tested significance using 9999 permutations, and used Bray-Curtis dissimilarity as our distance metric between years. Because the high-Arctic climate is highly variable, there may not necessarily be a linear trend in temperature or precipitation across years. This means that we should not expect a linear trend in activity periods across years. We therefore treat 'year' as a categorical variable and assume no more relationship between 1996 and 1997 than between 1996 and 2016. If the interaction between year and order was not significant, we removed this term and re-fit the PERMANOVA.

Unequal variance in activity periods among groups (orders or years) can cause false positive PERMANOVA results. To assess whether this is likely in our case, we tested whether variance among groups differed for any grouping that was significantly related to activity period in the PERMANOVAs. We calculated variance in activity periods using the R (R Core Team, 2016) function 'betadisper' from the package *vegan* (Oksanen *et al.*, 2019) and assessed whether this variance differed between groups using the function 'anova', also from *vegan*.

Section S9.2 Results

While some high-Arctic plants have extremely short flowering periods, most of the species in our dataset flowered for several weeks (Fig. S8). Two particularly important plants, *Salix arctica* (Malpighiales) and *Dryas octopetala* (Rosales), were consistently visited by more taxa than other plants. *Salix arctica* flowers early in the season while *Dryas octopetala* flowers through most of the season. Plant flowering periods were conserved within year and within order, but the interaction between the two was not significant ($F_{9,101}$=0.871, p=0.960). Re-fitting the PERMANOVA test without this interaction, plant flowering periods were conserved within orders and years ($F_{9,137}$=2.40, p=0.001 and $F_{4,137}$=2.73, p=0.001, respectively). Plant flowering periods were not differently variable across orders ($F_{9,141}$=1.84, p=0.067), meaning that the PERMANOVA result is a true positive. Plant flowering periods were differently variable across years ($F_{4,146}$=2.54, p=0.042), meaning that the PERMANOVA result may be a false positive.

Among pollinators, Diptera were more active than most other insects and were most active early in the season. This early peak is largely due to Chironomidae, which were highly active early in the season but rarely observed after week 28. Other major Diptera groups were most active in the middle of the season (Empididae) or equally active throughout the season

(Muscidae). Pollinator activity periods were conserved within order, year, and their interaction ($F_{3,312}$=3.37, p=0.001; $F_{4,312}$=4.46, p=0.001; and $F_{12,312}$=1.37, p=0.001, respectively). However, activity periods were differently variable across year-order combinations ($F_{19,312}$=10.7, p<0.001). This means that the PERMANOVA results may be unreliable. Re-fitting the PERMANOVA test without this interaction, plant flowering periods were conserved within orders and year ($F_{3,324}$=3.32, p=0.001 and $F_{4,324}$=4.40, p=0.001, respectively). Activity periods were not differently variable across orders ($F_{3,328}$=1.72, p=0.163) or years ($F_{4,327}$=1.86, p=0.177), meaning that the second PERMANOVA results are reliable.

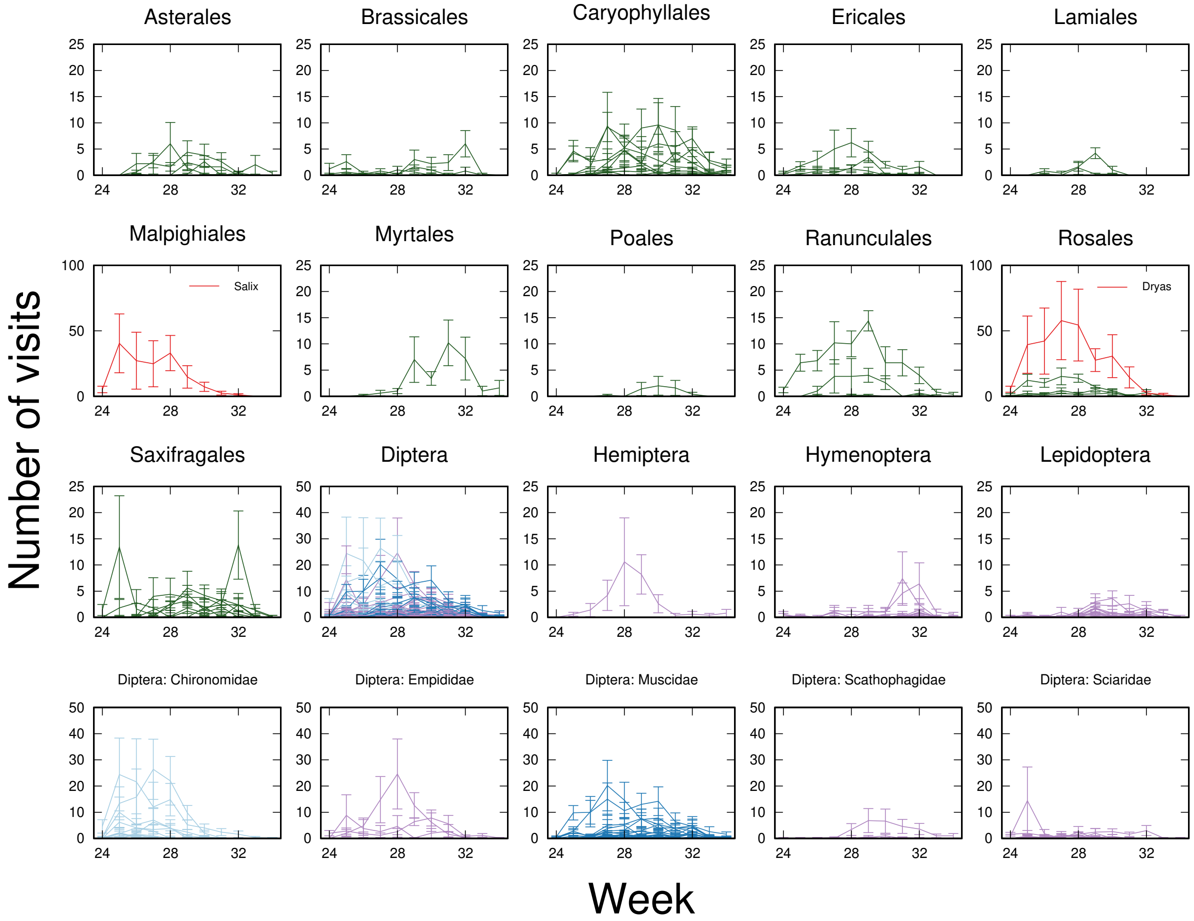

Figure S8: Mean activity periods (number of visits observed per week, across all five years) of plants and insects, grouped by order. Error bars indicate ±SE. Two key plants, *Salix arctica* (Malpighiales) and *Dryas octopetala* (Rosales), are highlighted in red. Among pollinators (Diptera, Hemiptera, Hymenoptera, and Lepidoptera), the Diptera were the most species-rich and most frequent flower visitors. To provide more detail, we show the activity periods of the five most species-rich Diptera families separately. Chironomidae (light blue) and Muscidae (dark blue) are highlighted in the main Diptera plot.

References

Aljanabi, S.M. & Martinez, I. (1997). Universal and rapid salt-extraction of high quality genomic DNA for PCR-based techniques. *Nucleic Acids Research*, 25, 4692–4693.

Folmer, O., Black, M., Hoeh, W., Lutz, R. & Vrijenhoek, R. (1994). Dna primers for amplification of mitochondrial cytochrome *c* oxidase subunit i from diverse metazoan invertebrates. *Molecular Marine Biology and Biotechnology*, 3, 294–299.

Oksanen, J., Blanchet, F.G., Friendly, M., Kindt, R., Legendre, P., McGlinn, D., Minchin, P.R., O'Hara, R.B., Simpson, G.L., Solymos, P., Stevens, M.H.H., Szoecs, E. & Wagner, H. (2019). *vegan: community ecology package*. R package edn.

R Core Team (2016). *R: a language and environment for statistical computing. Version 3.6.3*. R Foundation for Statistical Computing, Vienna, Austria.

Rasmussen, C., Dupont, Y.L., Mosbacher, J.B., Trjøelsgaard, K. & Olesen, J.M. (2013). Strong impact of temporal resolution on the structure of an ecological network. *PLoS ONE*, 8, e81694.

Ratnasingham, S. & Hebert, P.D.N. (2007). BOLD: The Barcode of Life Data System: Barcoding. *Molecular Ecology Notes*, 7, 355–364.

Schindelin, J., Arganda-Carreras, I., Frise, E., Kaynig, V., Longair, M., Pietzsch, T., Preibish, S., Ruedan, C., Saalfeld, S., Schmid, B., Tinevez, J.Y., White, D.J., Hartenstein, V., Eliceiri, K., Tomancak, P. & Cardona, A. (2012). Fiji: an open-source platform for biological-image analysis. *Nature Methods*, 9, 676–682.

Shokralla, S., Porter, T.M., Gibson, J.F., Dobosz, R., Janzen, D.H., Hallwachs, W., Golding, G.B. & Hajibabaei, M. (2015). Massively parallel multiplex DNA sequencing for specimen identification using an Illumina MiSeq platform. *Scientific Reports*, 5, 9687.

Skiljan, I. (2021). Irfanview version 4.59. *https://www.irfanview.com*, accessed Dec. 23, 2021.

Vesterinen, E.J., Puisto, A.I., Blomberg, A.S. & Lilley, T.M. (2018). Table for five, please: dietary partitioning in boreal bats. *Ecology and Evolution*, 8, 10914–10937.

Vesterinen, E.J., Ruokolainen, L., Wahlberg, N., Peña, C., Roslin, T., Laine, V.N., Vasko, V., Sääksjärvi, I.E., Norrdahl, K. & Lilley, T.M. (2016). What you need is what you eat? prey selection by the bat *Myotis daubentonii*. *Molecular Ecology*, 25, 1581–1594.

Wirta, H., Várkonyi, G., Rasmussen, C., Kaartinen, R., Schmidt, N.M., Hebert, P.D., Barták, M., Blagoev, G., Disney, H., Ertl, S., Gjelstrup, P., Gwiazdowicz, D.J., Huldén, L., Ilmonen, J., Jakovlev, J., Jaschhof, M., Kahanpää, J., Kankaanpää, T., Krogh, P.H., Labbee, R., Lettner, C., Michelsen, V., Nielsen, S.A., Nielsen, T.R., Paasivirta, L., Pedersen, S., Pohjoismäki, J., Salmela, J., Vilkamaa, P., Väre, H., von Tschirnhaus, M. & Roslin, T. (2016). Establishing a community-wide DNA barcode library as a new tool for arctic research. *Molecular Ecology Resources*, 16, 809–822.
